# Pleiotropic Potential of *Evernia prunastri* Extracts and Their Main Compounds Evernic Acid and Atranorin: In Vitro and In Silico Studies

**DOI:** 10.3390/molecules29010233

**Published:** 2023-12-31

**Authors:** Elżbieta Studzińska-Sroka, Magdalena Bulicz, Marika Henkel, Natalia Rosiak, Magdalena Paczkowska-Walendowska, Dominik Szwajgier, Ewa Baranowska-Wójcik, Katarzyna Korybalska, Judyta Cielecka-Piontek

**Affiliations:** 1 Department of Pharmacognosy and Biomaterials, Poznan University of Medical Sciences, Rokietnicka 3 Str., 60-806 Poznań, Poland; m.j.bulicz@gmail.com (M.B.); marikagomula@gmail.com (M.H.); nrosiak@ump.edu.pl (N.R.); mpaczkowska@ump.edu.pl (M.P.-W.); jpiontek@ump.edu.pl (J.C.-P.); 2 Department of Biotechnology, Microbiology and Human Nutrition, University of Life Sciences in Lublin, Skromna 8 Str., 20-704 Lublin, Poland; dominik.szwajgier@up.lublin.pl (D.S.); ewa.baranowska@up.lublin.pl (E.B.-W.); 3 Department of Patophysiology, Poznan University of Medical Science, Rokietnicka 8 Str., 60-806 Poznań, Poland; koryb@ump.edu.pl

**Keywords:** oak moss, lichen, enzyme inhibition, neurodegenerative diseases, molecular docking

## Abstract

*Evernia prunastri* is a lichen widely distributed in the Northern Hemisphere. Its biological properties still need to be discovered. Therefore, our paper focuses on studies of *E. prunastri* extracts, including its main metabolites evernic acid (EA) or atranorin (ATR). Phytochemical profiles using chromatographic analysis were confirmed. The antioxidant activity was evaluated using in vitro chemical tests and in vitro enzymatic cells-free tests, namely superoxide dismutase (SOD), glutathione peroxidase (GPx), glutathione reductase (GR), and catalase (CAT). The anti-inflammatory potential using cyclooxygenase-2 (COX-2) and hyaluronidase were determined. The neuroprotective potential using acetylcholinesterase, (AChE), butyrylcholinesterase (BChE), and tyrosinase (Tyr) was estimated. The hypoglycemic activity was also confirmed (α-glucosidase). Principal component analysis was performed to determine the relationship between the biological activity of extracts. The inhibitory effect of EA and ATR on COX-2 AChE, BChE, Tyr, and α-glucosidase was evaluated using molecular docking techniques and confirmed for EA and ATR (besides α-glucosidase). The penetration of EA and ATR from extracts through the blood–brain barrier was confirmed using the parallel artificial membrane permeability assay blood–brain barrier test. In conclusion, depending on chemical surroundings and the concentration, the *E. prunastri* extracts, EA or ATR, showed attractive pleiotropic properties, which should be further investigated.

## 1. Introduction

Age is one of the most significant causes of civilizational diseases [1]. Age-related health disorders can result from an increase in the level of reactive oxygen species (ROS), which affects the induction of inflammatory processes and also generates neurodegenerative changes associated with progressive neuronal damage [2,3]. Thus, one of the important medical problems is the emergence of neurodegenerative diseases. Neurodegenerative disorders (e.g., Alzheimer’s—AD or Parkinson’s disease—PD), although they differ in their clinical presentation, have numerous features in common. In their course, disorders of neurotransmission (cholinergic or dopaminergic) are observed, as well as an increased concentration of pathological proteins in the cytoplasm [4,5] contributing to neuronal damage. This results in an inflammatory process manifested by, among other things, the induction of oxidative stress and the influx of inflammatory cells into the central nervous system (CNS) [6]. Recent research has suggested that the occurrence of neurodegenerative diseases, particularly Alzheimer’s disease (AD), is associated with impaired glucose metabolism. Studies have shown that the risk of AD increases in patients with diabetes [7]. Blood glucose disturbances can negatively affect CNS function and impairment of autonomic nervous system neurons in the hypothalamus and brainstem in patients with type 2 diabetes [8]. Hyperglycemia and impaired blood insulin levels impair the neuroprotective properties of insulin in the central nervous system [9,10] and cause an overproduction of free radicals [11], which will damage nerve tissue and retinal vessels. Inflammation and high levels of free radicals are also a consequence of increasingly reported strokes.

Lichens are an interesting group of organisms whose existence depends on the coexistence of algal and fungal cells. This relationship results in the production of interesting substances with valuable biological potential [12]. Many scientific reports confirm the biological value of lichens and their metabolites. Recent work tests substances extracted from lichen husks in various research models and can prove their antioxidant, anti-inflammatory, anti-cancer, anti-diabetic, or neuroactive effects. However, the pleiotropic effects of lichen compounds, including those on the CNS, are still poorly described [13].

**Figure 1:**
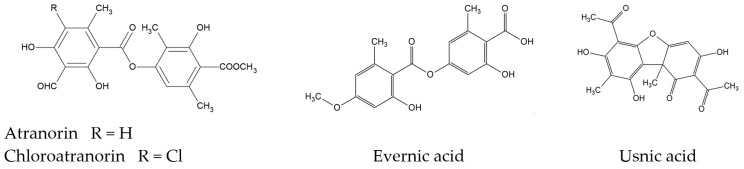
The chemical structures of the main secondary metabolites of *E. prunastri*.

*Evernia prunastri* (oakmoss) is a cosmopolitan lichen species recorded on all continents (except Antarctica) and is one of the most abundant lichen species in the Northern Hemisphere. The thallus of *E. prunastri* is rich in depsides (mainly evernic acid, atranorin, and chloroatranorin). The presence of the dibenzofuran derivative usnic acid was also detected [14,15] (Figure 1). Furthermore, Staples et al. (2020) report the presence of lecanoric, salazinic, and physodic acids in samples of *E. prunastri* thallus from Russia or the USA [16]. It should be emphasized that the relatively low molecular weight of lichen compounds and their lipophilicity suggest an ability to penetrate the blood–brain barrier [17]. The high content of compounds encourages research on the pleiotropic, including neuroprotective, potential of *E. prunastri*.

Little is known about the biological properties justifying the neuroprotective potential of *E. prunastri*. The methanolic extract of *E. prunastri* has been evaluated for its neuroprotective properties and cytoprotective effects against cells of the U373-MG and SH-SY5Y lines. Evernic acid showed similar effects [13]. The neuroprotective activity was demonstrated against atranorin present in *E. prunastri*, based on the growth of Neuro2A neurites, and no cytotoxic effects of atranorin were observed in the model tested. In addition, atranorin modulates the expression of genes encoding the neurotrophic markers BDNF and NGF [18]. There are also reports of atranorin’s ability to inhibit protein tyrosine phosphatase (PTP1B), resulting in lower cellular glucose concentrations [19]. The antioxidant activity of extracts and compounds from *E. prunastri* tested by the DPPH reagent method indicated average antioxidant activity even in low doses of the extract [14,20]. In addition, the evernic acid present in the acetone extract showed good permeability across the blood–brain barrier (BBB) in the parallel artificial membrane permeability assay (PAMPA) [21]. This indicates that further studies on extracts of other polarities, as well as on evernic acid and their biological properties, are necessary.

It was hypothesized that *E. prunastri* may provide extracts and compounds with valuable properties for the prevention and therapy of civilizational diseases as well as CNS diseases. Therefore, this study aims to evaluate the pleiotropic properties of different polarity extracts from *E. prunastri* as well as evernic acid and atranorin, the main secondary metabolites of *E. prunastri*.

## 2. Results and Discussion

### 2.1. Phytochemicals Analysis of Evernia prunastri

#### 2.1.1. High-Performance Liquid Chromatography (HPLC) Analyses

**Figure 2:**
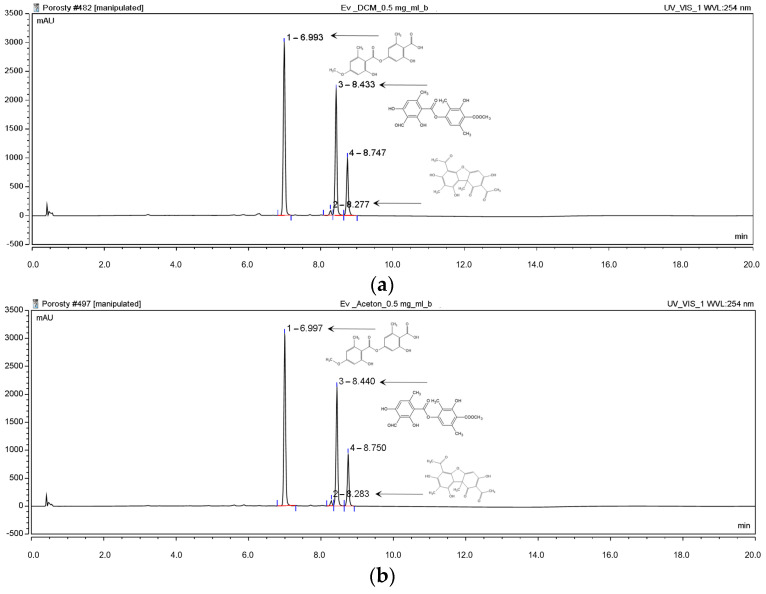

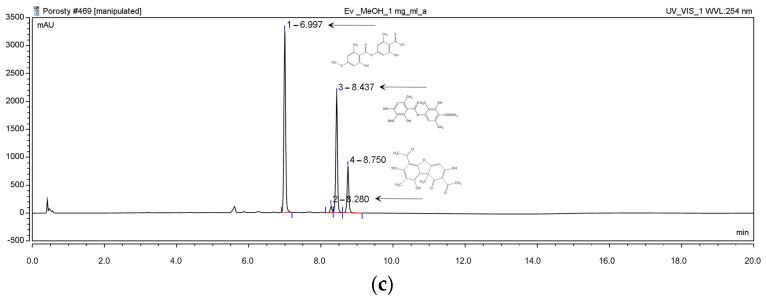
The chromatogram of extracts from *Evernia prunastri* shows the identified compounds, i.e., evernic acid, atranorin, and (+)-usnic acid, respectively. The compounds were characterized by Rt = 6.99 min, 8.44 min, and 8.28 min, respectively (Rt—retention time): dichloromethane (0.5 mg/mL) (**a**), acetone (0.5 mg/mL) (**b**), and methanol (1 mg/mL) (**c**).

The HPLC chromatographic analysis was performed to determine the active compounds’ content in the tested extracts from *E. prunastri*. For this purpose, the gradient elution method (acetonitrile and formic acid 0.5%) was performed. The content of evernic acid, atranorin, and usnic acid was assessed (Figure 2) (evernic acid Rt = 6.99 min, atranorin Rt = 8.44 min, and usnic acid Rt = 8.28 min). The obtained results indicated that tested extracts differ in terms of the content of individual substances. The hexane extract (Hex extract) contained the most atranorin (61.04%) and (+)-usnic acid (5.25%). The most abundant substance in evernic acid was the dichloromethane extract (DCM extract) (63.13%). All results are presented in Table 1. The chromatograms of Hex extract, methanol-water extract (MeOH-H_2_O extract), and water extract (H_2_O extract) are presented in Supplementary Materials (Figures S1–S3).

#### 2.1.2. Total Polyphenol Content

Polyphenols are a group of biologically active compounds whose therapeutic and preventive effects have been proven by numerous studies [22]. The antioxidant properties of these compounds are attributed to the ability to neutralize ROS, which causes the development of many civilization diseases, including CNS diseases [23]. Lichens are a rich source of lichen-derived polyphenols. This suggests that the assessment of the content of these compounds in extracts provides important input data on the tested samples.

**Figure 3:**
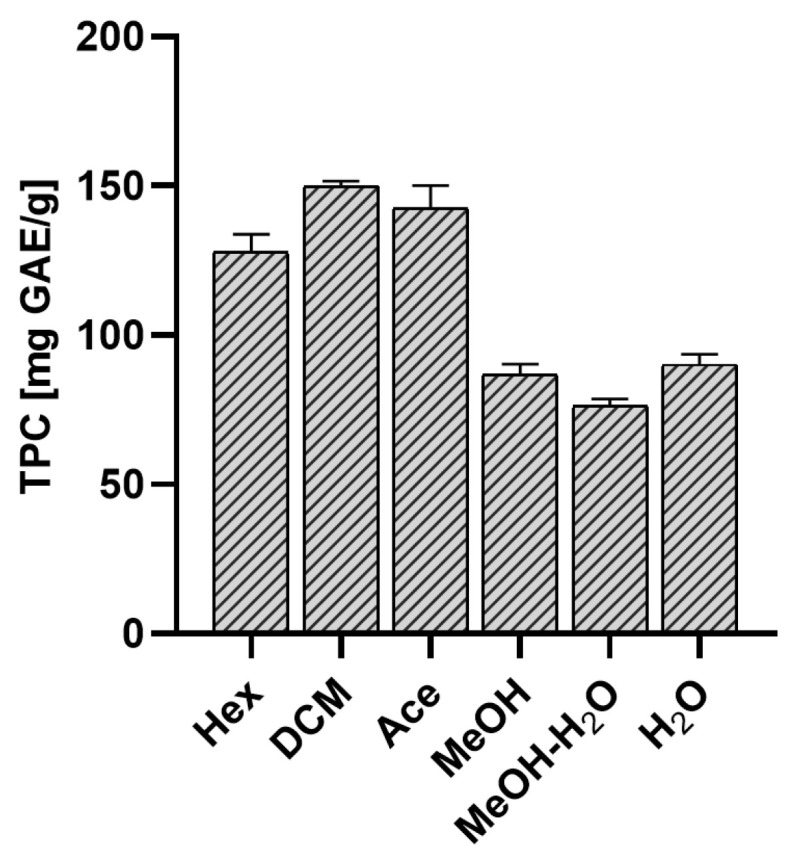
The polyphenolic content in *Evernia prunastri* extracts expressed as mg gallic acid equivalent/g of extract (GAE/g of extract). The mean values ± SD from n = 6 measurements are presented. Hex—hexane extract, DCM—dichloromethane extract, Ace—acetone extract, MeOH—methanol extract, MeOH-H_2_O—methanol-water extract, H_2_O—water extract.

Results of our experiments indicate that *E. prunastri* extracts are a rich source of polyphenols. Extracts with the lowest polarity were richer in polyphenols (DCM extract > Ace extract > Hex extract) than those that were more polar (Figure 3).

Our results are consistent with the reports of other authors. Aoussar et al. (2020) [20] examined acetone extracts obtained by 24-h maceration, prepared from *E. prunastri* thallus collected at different times of the year. The content of polyphenols was similar to our analysis [20]. In another work, the author examined polyphenols’ content in dichloromethane, acetone, and methanol extracts from *E. prunastri*. Aoussar et al. (2017) noted the highest polyphenol content in the acetone extract (163 ± 26.45 µg GAE/mg of dry extract), followed by the dichloromethane extract (128.66 ± 3.75 µg GAE/mg of dry extract) and MeOH (94.33 ± 24.82 µg GAE/mg of dry extract), which is also similar to our results [24]. The MeOH extract from *E. prunastri* was also tested by Mitrović et al. (2011), with results similar to ours [25].

### 2.2. Biological Activity of Evernia prunastri

#### 2.2.1. Antioxidant Activity

Homeostasis of biochemical processes occurring in the CNS is crucial for proper brain functioning. Oxidative stress, manifested by excessive production of free radicals and insufficient level of their elimination, disturbs this balance necessary for health [26]. Uncontrolled ROS excess results in brain macromolecule damage. It could also be the cause of inflammation of different tissues, as well as those of the brain. It, in turn, leads to diseases of the nervous system [27]. Therefore, antioxidant activity is important in protecting the balance of the human body, including the CNS.

##### 2,2-Diphenyl-1-Picrylhydrazyl (DPPH) and Cupric Ion Reducing Antioxidant Capacity (CUPRAC) Assays

Due to the well-confirmed involvement of ROS in the initiation of the inflammatory process [28], which accompanies different civilization diseases, including neurodegenerative degeneration [28,29], we undertook research on the antioxidant potential of *E. prunastri* and evernic acid extracts.

In the first stage of research, the antioxidant activity of *E. prunastri* extracts was examined using in vitro chemical analyses (DPPH and CUPRAC). The first one determines the ability of tested substances to scavenge free radicals. The second one determines the ability to reduce copper ions (Cu^2+^). The obtained results suggest that the antioxidant activity of the tested extracts varies (Table 2). The highest anti-free radical ability is demonstrated by extracts with higher polarity: methanol extract (MeOH extract), methanol-water extract (MeOH-H_2_O extract), and water extract (H_2_O extract). Extracts obtained using hexane, dichloromethane, or acetone showed low activity. An inverse relationship was observed in the case of the CUPRAC analysis: the highest activity was observed in hexane, dichloromethane, and acetone extracts (Table 2).

Previous discussions and analysis of the polyphenol content, which complemented the HPLC analysis of the tested extracts, indicated that a higher polyphenol content characterizes extracts with lower polarity. From the results of the DPPH and CUPRAC experiments, it appears that lichen polyphenols (secondary metabolites: atranorin, usnic acid, and evernic acid), which dissolve well in a weakly polar medium, possess a low ability to scavenge the DPPH radical. However, they play a significant role in the reduction in copper ions, as indicated by the low concentrations required to induce the IC_0.5_ effect.

The antioxidant activity of extracts from the thallus of various lichen species, including *E. prunastri*, as well as their compounds, has been previously described in the literature [13,30,31,32]. Kosanić et al. (2013) found that the acetone extract of *E. prunastri* had a weaker ability to scavenge DPPH free radicals compared to evernic acid [14]. This activity was also found to be weaker than the activity of another species tested at the same time, which contained physodic acid—a strong antioxidant found in lichens. Studzińska-Sroka et al. (2021) evaluated the antioxidant activity of acetone extract from *E. prunastri*, *C. uncialis*, and *P. sulcata*. Compared to the other two extracts, *E. prunastri* had the weakest effect [21].

##### Chelating Activity of Fe^2+^ and Cu^2+^

Neurodegenerative diseases are related to the appearance of chronic oxidative stress and protein aggregation. The literature indicates that these pathological processes involve the contribution of metal ions [33]. Research has shown that transition metal ions, specifically Cu(I/II), Zn(II), and Fe(II/III), play a significant role in neuro-transmission [34]. This finding further emphasizes the importance of research in this area and highlights the potential for metal ions to be used as a therapeutic target in the development of drugs for neurodegenerative diseases. Although there are conflicting reports on the function of Cu(II) in AD, most studies indicate a higher level in AD [35] and their significance in inducing amyloid-β [36] or increasing amyloid-β toxicity [37]. Therefore, metal ions are increasingly considered a therapeutic target when designing drugs used in neurodegeneration [35,38].

Therefore, our analysis of biological activity included tests for the chelating ability of metal ions. Results presented in Table 3 and Table 4 indicate that all the extracts of *E. prunastri* can chelate Fe^2+^ and Cu^2+^ ions in a dose-dependent manner. Analysis of the results indicates that the polar extracts possess the ability to chelate Fe ions. The highest activity was found in MeOH-H_2_O and H_2_O extracts. The Hex extract with a high concentration of atranorin also showed high activity. Extracts that contained significant amounts of evernic acid had a weak effect, suggesting that this compound may not affect the tested property. In terms of the ability to chelate copper ions, all *E. prunastri* extracts had a relatively even effect, with over 80% chelation of Cu^2+^ at the highest tested concentration (Table 4).

Previous literature indicates that plant polyphenols can chelate metal ions [39]. Little is known about the ability of multi-component lichen extracts and their metabolites to bind transition metals. Research in the field of lichenology reports a diverse ability to adsorb metal ions (including Cu^2+^ and Fe^2+^/Fe^3+^) by depsidones and depsides of *H. physodes* (*Parmeliaceae* family), including by atranorin, often present in the thalli of lichens [40]. In addition, he wrote about the ability of lichen extracts to bind Fe ions. The potential of acetate and methanol extracts from various lichen species (including *Cladonia foliacea*, *Cladonia rangiferaina*, *Flavoparmelia caperata*, and *Xanthoria parietina*) were evaluated in other studies [41,42]. In almost each of the cases, the extract with higher polarity was more active.

##### Effect on Antioxidant Enzyme Activity

The human body has the ability to counter the excess of free radicals by producing antioxidant enzymes, such as superoxide dismutase (SOD), catalase (CAT), glutathione peroxidase (GPx), or glutathione reductase (GR) [43]. The role of enzymatic antioxidants varies and includes breaking down superoxide anions into less harmful ROS (SOD), reducting H_2_O_2_ to H_2_O and molecular O_2_ (CAT, GPx) [44]. At the same time, the role of GR is to recycle oxidized glutathione [45].

Our research showed that extracts from *E. prunastri* are capable of extracts inhibiting the activity of antioxidant enzymes, which suggests that they may reduce the efficiency of the body’s defence mechanisms against oxidative stress. Among the tested enzymes, CAT was found to be the least sensitive to the effects of substances extracted from lichens, including Ace extract, MeOH extract, and evernic acid. However, these substances showed a slight ability to inhibit SOD (34.1% ± 1.7), GR (10.3% ± 3.7), and GPx (10.0% ± 2.6). These results are consistent with previous findings that suggest a slight effect of evernic acid on inhibiting the activity of antioxidant enzymes (GPx 20.0% ± 2.1, GR—no activity, and SOD 21.2% ± 0.0) [21]. The results are presented in Table 5.

The influence of various lichen substances on the activity of antioxidant enzymes has been previously studied but most of the research has used tissue material. Previous experiments have revealed that usnic acid enhances the effectiveness of the antioxidant system by improving the functions of superoxide dismutase (SOD) and glutathione synthase in hippocampal cells [46]. On the other hand, methanol extracts from *Cetraria islandica* have been shown to increase the activities of SOD and GPx [47]. Therefore, more experiments are needed to assess whether substances from *E. prunastri* will affect intracellular antioxidant systems under physiological conditions.

#### 2.2.2. Anti-Inflammatory Activity

##### Effect on Cyclooxygenase-2 Activity (COX-2)

While COX-1 is a constitutive isoform and is involved in maintaining homeostasis in the body, COX-2 is activated during the inflammatory response. An increase in the activity of this enzyme is also observed in the case of degenerative changes and cancer [48]. Moreover, studies have shown increased COX-2 expression in neuroinflammatory conditions [49] and neurodegenerative diseases [50]. Thus, inhibition of COX-2 activity has become a critical target of many drugs [51]. It has been proven that inflammation of the nervous system is one of the main features of AD [29]. Epidemiological studies have shown that using anti-inflammatory drugs reduces the risk of AD, which is one of the most common types of dementia in elderly people [52].

The results we present indicate that *E. prunastri* extracts can inhibit COX-2. While the level of inhibition varied, the MeOH extract had the strongest inhibitory effect (96.3% ± 1.9%). Our analysis suggests that extracts rich in evernic acid (DCM, Ace, and MeOH) had the strongest impact among all the tested samples. Furthermore, we found that evernic acid itself was able to inhibit the enzyme to a significant extent, in contrast to atranorin, which according to our previous research was inactive (Table 6).

Studies have shown that lichen metabolites contain biologically active compounds that possess anti-COX-2 activity [53,54,55]. Based on the results of in vivo studies, lichen extracts can inhibit the inflammatory response in animal tissue [56]. The present work supports our previous findings, which demonstrated the activity of *E. prunastri* depside. The hexane extract rich in atranorin showed low activity, suggesting no COX-2 inhibitory potential. This conclusion was consistent with our prior research [57].

**Table 6:** Inhibition of cyclooxygenase-2 (COX-2) enzyme by the extracts of *Evernia prunastri* and evernic acid expressed as a % of inhibition and acetylsalicylic acid equivalent mg/mL.

Tested Sample	COX-2 Inhibition (%)	Acetylsalicylic Acid Equivalent (mg/mL)
Hex	13.7 ± 1.6	7.0 ± 0.4
DCM	44.8 ± 2.6	10.6 ± 0.5
Ace	57.5 ± 2.2	12.0 ± 0.4
MeOH	96.3 ± 1.9	16.5 ± 1.0
MeOH-H_2_O	12.4 ± 2.3	6.8 ± 0.9
H_2_O	18.5 ± 2.5	7.5 ± 0.2
EA	43.5 ± 2.7	10.4 ± 0.2
ATR	na*	na*

The mean values ± SD are presented. Hex—hexane extract, DCM—dichloromethane extract, Ace—acetone extract, MeOH—methanol extract, MeOH-H_2_O—methanol-water extract, H_2_O—water extract, EA—evernic acid, ATR—atranorin; “na*”—the value taken from the literature [57].

##### Anti-Hyaluronidase Activity

Hyaluronic acid is one of the most important components of the extracellular matrix (ECM) with diverse biological roles that depend on the chain length or molecular weight. Low molecular weight hyaluronic acid fragments (200–250 kDa) possess pro-inflammatory properties and play a significant role in initiating the inflammatory response [58]. Hyaluronic acid metabolism largely depends on the activity of hyaluronidase, which is responsible for breaking down hyaluronic acid chains. Hyaluronic acid is also a major component of the ECM in the human brain [59]. The presence of short-chain hyaluronic acid fragments may be important for the occurrence of inflammatory processes in the brain. Inhibition of hyaluronidase can reduce the concentration of low molecular weight hyaluronic acid and enzyme inhibitors may be helpful in the prevention and treatment of neurodegenerative changes [60].

**Figure 4:**
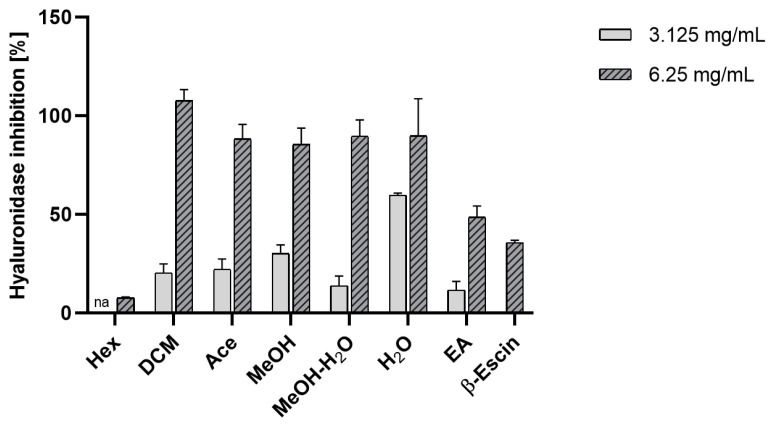
Inhibition of hyaluronidase by the extracts of *Evernia prunastri.* The mean values expressed as a % of inhibition ± SD from n = 3 are presented; “na”—not active hexane extract at concentration 3.125 mg/mL; Hex—hexane extract, DCM—dichloromethane extract, Ace—acetone extract, MeOH—methanol extract, MeOH-H_2_O—methanol-water extract 1:1 *v*/*v*, H_2_O—water extract, EA—evernic acid.

Our results, presented in Figure 4, indicate that extracts obtained from *E. prunastri* exhibit dose-dependent hyaluronidase inhibitory activity. The enzyme inhibition levels ranged from approximately 86 to 100% at the highest concentration tested. Further analysis of the results obtained at the 3.125 mg/mL concentration shows that the water extract of *E. prunastri* exhibited high activity. β-Escin, known for its hyaluronidase-inhibiting effect [61], tested at a concentration of 6.25 mg/mL, had a weaker effect than most of the tested extracts from *E. prunastri* and evernic acid.

In our previous studies, we also reported that lichen extracts have the ability to inhibit hyaluronidase [57]. We proved that the activity of these extracts and lichen compounds was higher than the standards we analyzed in parallel. Our results showed that evernic acid has a moderate ability to inhibit hyaluronidase (IC_50_ ~0.6 mg/mL of tested sample at 6.0 mg/mL prepared for tests) [21]. The activity of the extracts is likely due to the presence of evernic acid as the inhibition activity was the highest in the DCM extract, which has the highest content of this compound. Galanty et al. (2021) also indicated the ability of usnic acid to inhibit hyaluronidase, similar to quercetin [62]. The Hex extract containing ~5% of usnic acid was the least active among our extracts.

#### 2.2.3. Neuroprotective Activity

##### Effect on Acetylcholinesterase (AChE) and Butyrylcholinesterase (BChE) Activity

Acetylcholine is an important neurotransmitter responsible for attention, memory, and motivation [63]. Both acetylcholinesterase (AChE) and butyrylcholinesterase (BChE) are responsible for acetylcholine metabolism, therefore regulating its level in healthy tissue and ensuring appropriate neurotransmission. In AD, the concentration of acetylcholine is reduced due to activity of cholinesterases, which exacerbates the symptoms of the disease [64]. There is a shift towards increased expression of BChE. Therefore, cholinesterase inhibitors are currently used in the treatment of AD [65]. Finding natural molecules that can inhibit these enzymes could be helpful in developing effective drugs for treating this type of dementia.

**Figure 5:**
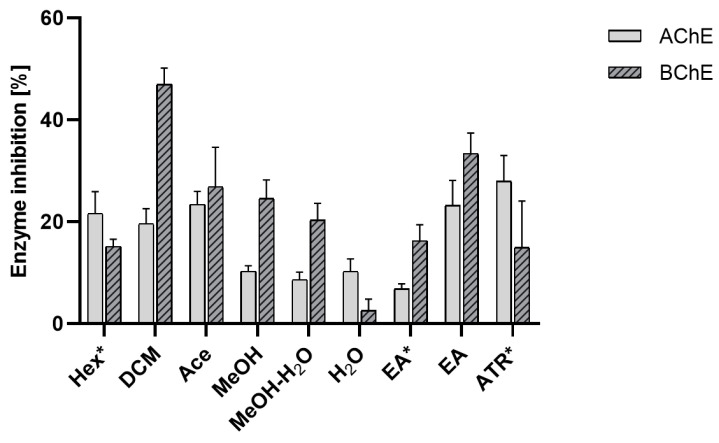
Inhibition of acetylcholinesterase (AChE) and butylcholinesterase (BChE) by the extracts of *Evernia prunastri.* The mean values expressed as a % of inhibition ± SD from n = 4 measurements for extracts and n = 3 measurements for lichen metabolites are presented; “*”—samples measured at concentration 10 mg/mL; Hex—hexane extract, DCM—dichloromethane extract, Ace—acetone extract, MeOH—methanol extract, MeOH-H_2_O—methanol-water, H_2_O—water extract, EA—evernic acid, ATR—atranorin.

Our results showed that the extracts from *E. prunastri* exhibit differential anti-AChE and anti-BChE activity, with a predominantly greater inhibitory effect on BChE. The exception is the Hex extract, which is characterized by a high content of atranorin and, like this depside, inhibits AChE more strongly. On the other hand, the inhibitory activity on cholinesterase of DCM, Ace, MeOH, and MeOH-H_2_O extracts seems to be dependent on the presence of both evernic acid and atranorin (Figure 5). It should be noted that the strong inhibition of BChE by *E. prunastri* substances is a beneficial phenomenon because BChE is present in higher concentrations than AChE in AD patients [66]. In addition, Guillozet et al. (1997) proved that BChE might initiate the transformation of Aβ from an initially benign substance to a potentially harmful one [67]. Thus, higher inhibition of BChE would more beneficial. Among the tested extracts, only the water extract showed stronger inhibition of AChE than BChE. This could be attributed to the extract’s different phytochemical profile. On the other hand, the Hex extract with a high content of atranorin did not exhibit any inhibitory effect on AChE.

As AD is an increasing social issue, it is important to analyze the biological activity of lichen toward their inhibition of cholinesterases. Our findings are consistent with the results obtained by other researchers. Cakmak and Gülçin (2019) proved the anticholinergic activity of usnic acid [68]. Activity towards AChE was confirmed by Nagar et al. (2023) [69]. Other groups have reported the AChE-inhibiting effect of perlatoric acid [18,69]. Moreover, caperatic acid, an aliphatic molecule with anticholinergic potential found in *P. glauca*, also had the ability to inhibit cholinesterases [70].

##### Effect on Tyrosinase Activity

Tyrosinase is a metalloenzyme involved in regulating the production of melanin in the human body [71]. This enzyme converts L-tyrosine and L-DOPA to o-dopaquinone [72], which is further transformed into melanin and other polyphenolic compounds [71]. In humans, tyrosinase is present in the eyes, ears, and skin [73]. Its presence has also been detected in the substantia nigra of the brain, where it is responsible for the production of neuromelanin. Studies have shown that intracellular neuromelanin levels [74], as well as the presence of dopamine-quinones, which increase the level of ROS [75], may be related to the initiation of Parkinson’s disease.

**Figure 6:**
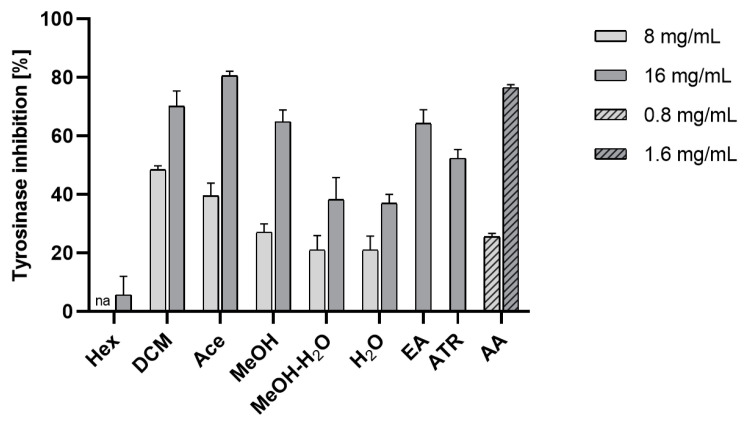
Inhibition of tyrosinase by the extracts of *Evernia prunastri.* The mean values expressed as a % of inhibition ± SD from n = 6 measurements for extracts and azelaic acid and n = 3 for evernic acid and atranorin are presented; “na”—not active hexane extract at concentration 0.8 mg/mL; Hex—hexane extract, DCM—dichloromethane extract, Ace—acetone extract, MeOH—methanol extract, MeOH-H_2_O—methanol-water extract, H_2_O—water extract, EA—evernic acid, ATR—atranorin, AA—azelaic acid.

Our research on *E. prunastri* has proven that extracts obtained from the lichen’s thalli have the ability to inhibit tyrosinase. The extracts with the highest inhibitory activity are DCM, Ace, and MeOH, which have a high content of evernic acid and atranorin. Evernic acid and atranorin itself also showed the potential to inhibit tyrosinase. It is worth emphasizing that evernic acid-rich extracts and evernic acid and atranorin had a similar anti-tyrosinase effect compared to azelaic acid tested in 10 times lower concentrations (azelaic acid is used in hyperpigmentary disorders [76]) (Figure 6). However, it is interesting that the Hex extract rich in atranorin had the weakest effect compared to its active depside. It can be related to the antagonistic activity of compounds from Hex extract.

Previous scientific research has reported the ability of lichen extracts to inhibit tyrosinase. Bhaskar et al. (2009) discovered the inhibition of this enzyme by methanol extracts of lichens from the *Graphidaceae* family [77]. Similarly, methanol extracts of cultured symbionts of three different lichen species *A. awasthii, H. podocarpa,* and *P. tinctorum* also displayed tyrosinase inhibitory activity [78]. Additionally, depsidones, fumaroprotocetraric acid [79], and physodic acid [57] also had the ability to inhibit the enzyme. The tyrosinase-inhibiting activity of extracts containing evernic acid and the compound itself was assessed for the first time. The discovered activity suggests the possibility of use in treating diseases with increased tyrosinase expression and encourages further work in this area.

##### α-Glucosidase Inhibitory Assay

α-Glucosidase is an enzyme that plays a key role in breaking down complex carbohydrates into monosaccharides, which directly affects the glucose level in our bloodstream [80,81]. Recent studies have shown a correlation between the likelihood of developing AD and risk factors such as diabetes and obesity [82]. Moreover, scientists have suggested an association between hyperglycemia and the progression of PD [83].

Due to the role of hyperglycemia in the development of civilization diseases (e.g., cancer, cardiac dysfunction, and diabetes) [84,85] as well as nervous system dysfunctions [86], we examined the effect of *E. prunastri* extracts on α-glucosidase activity. Our findings showed that MeOH-H_2_O and H_2_O extracts had almost no inhibitory effect on the enzyme. However, Hex extract, which is rich in atranorin, showed an important inhibitory level, even at lower concentrations. At the concentration of 3 mg/mL, this extract inhibited glucosidase almost four times stronger (80.69%) than the other extracts tested at the same concentration (<~18%). The activity for Hex, DCM, Ace, and MeOH extracts was dose-dependent, with activity being higher at ~84% when the concentration was increased to 5 mg/mL. Under the experimental conditions, acarbose, an antidiabetic agent used to treat diabetes [87], had a comparable effect to *E. prunastri* extracts tested at 10 times lower concentrations (Table 7). The ability of lichen extracts and their metabolites to inhibit glucosidase is not well described compared to other biological tests. However, recent reports indicate that certain lichen-derived compounds, such as 3,5-dihydroxy-4-methoxylbenzoic acid, may inhibit the α-glucosidase (3,5-dihydroxy-4-methoxylbenzoic acid) [88]. Usnic acid has been shown to have a high level of such activity [89]. This may influence the high activity of the tested hexane extract. Duong et al. (2020) [90] and Karunaratne et al. (2014) [91] suggested that atranorin does not inhibit the enzyme, which supports the hypothesis that additional substances determine the activity of the extract. Our results clearly indicate that the main secondary metabolite of *E. prunastri*, evernic acid, strongly inhibits the enzyme. This suggests that extracts with high levels of evernic acid may have inhibitory activity. To the best of our knowledge, the activity of evernic acid against α-glucosidase has been determined for the first time.

### 2.3. Principal Component Analysis (PCA)

Principal component analysis (PCA) is an increasingly popular data analysis method used in many scientific disciplines [92]. PCA allows the observation of regularities among the examined variables [93]. The observed relationships are visualized in charts, which facilitates data interpretation.

**Figure 7:**
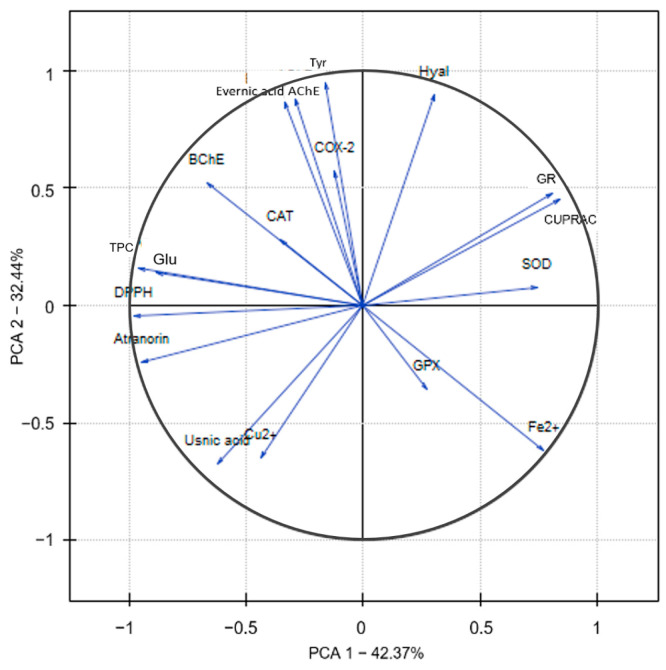
The relationship of *Evernia prunastri* extracts on the factorial plane formed by the first two principal components, where individual varieties were denoted as extracts—hexane, dichloromethane, acetone, methanol, methanol-water, and water; AChE—acetylcholinesterase inhibition, Atranorin—atranorin content, BChE—butyrylcholinesterase inhibition, CAT—catalase inhibition, COX-2—cyclooxygenase-2 inhibition, CUPRAC—CUPRAC analysis, Cu^2+^—chelating activity of Cu^2+^, DPPH—DPPH analysis, Evernic acid—evernic acid content, Fe^2+^—chelating activity of Fe^2+^, Glu—α-glucosidase inhibition, GPX—glutathione peroxidase inhibition, GR—glutathione reductase inhibition, Hyal—hyaluronidase inhibition, SOD—superoxide dismutase inhibition, TPC—total phenolic content, Tyr—tyrosinase inhibition, Usnic acid—usnic acid content.

Figure 7 shows the results of the PCA analysis for the extracts from *E. prunastri*, differentiated by polarity and analyzed for biological activity and the content of selected secondary metabolites. The first two principal components (PC) explained 74.81% of the total variability, with 42.37% for PC1 and 32.44% for PC2. PC1 showed a positive correlation with the inhibition of antioxidant enzymes (SOD, GR, and GPx) and the ability to inhibit hyaluronidase and reduce copper ions. The negative correlation between the studied variables, such as the total polyphenols content; cholinesterase inhibition; COX-2, CAT, and glucosidase inhibition; and the ability to scavenge free radicals. Additionally, it was also negatively correlated with the ability to chelate Cu^2+^ ions. On the other hand, PC2 showed a positive correlation with the ability variables describing the antioxidant potential and glucosidase inhibition. Conversely, it was negatively correlated with variables responsible for anti-inflammatory activity (hyaluronidase, COX-2), cholinesterase inhibitory activity, tyrosinase inhibitory activity, SOD and GR enzyme inhibition, and the ability to reduce Cu^2+^ ions (CUPRAC).

The results of the analysis also showed that there is a positive correlation in among the variables describing the activity of inhibiting enzymes BChE, AChE, Tyr, and COX-2, whose increased activity is noted in neuroinflammatory diseases [94]. Moreover, a strong correlation occurs between the variables BChE, AChE, Tyr, and COX-2 and the evernic acid. This suggests that the content of the main metabolite of *E. prunastri* may significantly impact the parameters that are sought for the prevention and treatment of neurodegenerative diseases. At the same time, the total polyphenol content is strongly correlated with anti-free radical activity and glucosidase inhibition, which are determined by the atranorin content. It is important to note that the inhibition of the antioxidant enzymes SOD, GPx, and GR is moderately or strongly negatively correlated with the TPC content, which indicates that polyphenols, as strong antioxidants, do not adversely affect cellular protection against antioxidant stress. However, such a correlation was not observed for CAT, which catalyzes the decomposition of H_2_O_2_ into oxygen and water [95].

**Figure 8:**
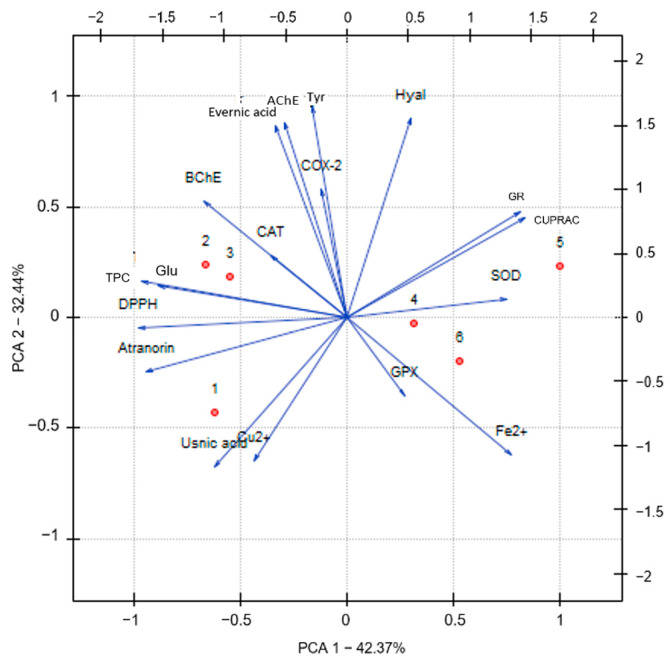
Principal component analysis (PCA) showing the factor loading plot, where individual varieties were denoted as extracts—hexane (1), dichloromethane (2), acetone (3), methanol (4), methanol-water (5), and water (6); AChE—acetylcholinesterase inhibition, Atranorin—atranorin content, BChE—butyrylcholinesterase inhibition, CAT—catalase inhibition, COX-2—cyclooxygenase-2 inhibition, CUPRAC—CUPRAC analysis, Cu^2+^—chelating activity of Cu^2+^, DPPH—DPPH analysis, Evernic acid—evernic acid content, Fe^2+^—chelating activity of Fe^2+^, Glu—α-glucosidase inhibition, GPX—glutathione peroxidase inhibition, GR—glutathione reductase inhibition, Hyal—hyaluronidase inhibition, SOD—superoxide dismutase inhibition, TPC—total phenolic content, Tyr—tyrosinase inhibition, Usnic acid—usnic acid content.

Figure 8 illustrates the relationship between the polarity of the reagent used to prepare the extract. The results allow us to distinguish two visible trends. Samples from low- and medium-polarity extracts are located close to each other and far from the group of reagents with higher polarity (MeOH extract, MeOH-H_2_O extract, and H_2_O extract). This suggests that the polarity of the solvent used affects the analyzed parameters.

### 2.4. Molecular Docking Study

Molecular docking techniques could be perceived ambiguously among researchers. However, it is also a technique helpful in the pharmaceutical industry’s design of new drugs [96]. In addition the studies emphasize that molecular docking results can be consistent with analyses carried out using in vitro methods [70].

Based on the molecular docking study, the inhibitory effect of evernic acid and atranorin against COX-2 (selected as a model protein in the area of anti-inflammatory activity), AChE, BChE, tyrosinase, and α-glucosidase (chosen for the area of neuroprotective activity) was evaluated. The active compounds were docked into the binding pocket of the enzyme using the known location of the active site (see Section 3.8. Molecular Docking). The best binding pose (i.e., orientation and geometry) was chosen based on the estimated lowest binding energy.

Identification and evaluation of the enzymes’ active site, their binding energies, the estimated inhibition constant (K_i_), and the bonds involved in the ligand–protein interaction were conducted. Calculated binding energy, estimated inhibition constant, and receptor contact are summarized in Table S1. Based on 3D models generated by Prank-Web (Table 8), it is confirmed that evernic acid and atranorin are in the active site of a given enzyme and interact with the active site’s residues.

#### 2.4.1. Cyclooxygenase-2 (COX-2)

**Figure 9:**
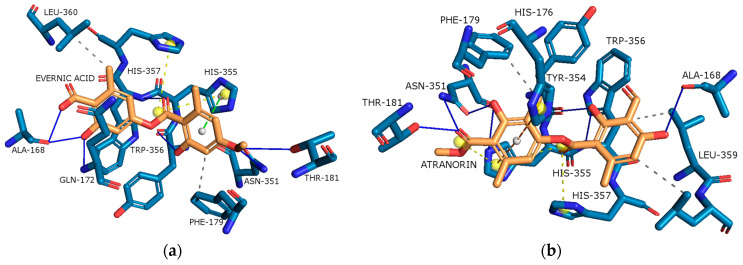
Proposed binding mode of evernic acid with COX-2 (PDB id: 5F1A). The key interactions of evernic acid with residues in the active sites of COX-2: hydrogen bonds (blue solid lines), hydrophobic interactions (grey dashed lines), π-stacking (green dashed line), and a salt bridge (yellow dashed line) (**a**); Proposed binding mode of atranorin with COX-2 (PDB id: 5F1A). The key interactions of atranorin with residues in the active sites of COX-2: hydrogen bonds (blue solid lines), hydrophobic interactions (grey dashed lines), π-stacking (green dashed line), and salt bridges (yellow dashed line) (**b**).

Figure 9a,b reveals the successful binding of evernic acid and atranorin to the active pocket of COX-2, respectively. In the case of evernic acid, the proposed binding mode showcases hydrogen bonds with Ala168, Gln172, Thr181, Trp356, and His357, as well as hydrophobic interactions with Phe179 and Leu360, π-stacking interactions with His355, and a salt bridge interaction with His357 and His355. The corresponding binding energy is determined to be −11.85 kcal/mol.

For atranorin, the suggested binding mode reveals hydrogen bonds with Ala168, Thr181, Asn351, Tyr354, and His355, along with hydrophobic interactions involving Phe179 and Leu359. Additionally, a salt bridge interaction with His355 and His357 was confirmed. The calculated binding energy was found to be −11.85 kcal/mol.

In addition, the docking scores for evernic acid and atranorin (binding energies −11.85 kcal/mol and −12.47 kcal/mol, respectively) have statistically significant differences (*p* ≤ 0.05). Obtained results suggest that atranorin is a more active compound against COX-2 than evernic acid.

The data obtained using molecular docking confirmed the in vitro results that ever-nic acid can inhibit COX-2. However, in contrast to the in vitro method, in silico studies of atranorin activity on COX-2 showed that atranorin inhibits the enzyme. The literature data on the anti-inflammatory activity of atranorin are scarce and indicate a dose-independent inhibitory effect on COX-2 [97]. Taking into account the preliminary character of our work, further experiments should be carried out to determine the anti-inflammatory activity of atranorin.

#### 2.4.2. Acetylcholinesterase (AChE)

**Figure 10:**
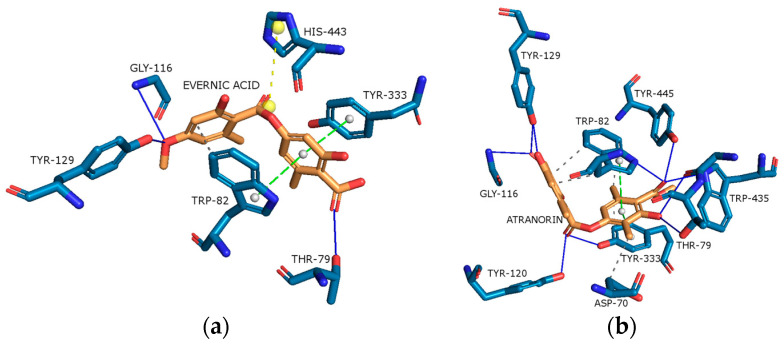
Proposed binding mode of evernic acid with AChE (PDB id: 4BDT). The key interactions of evernic acid with residues in the active sites of AChE: hydrophobic interactions (grey dashed lines), hydrogen bonds (blue solid lines), π-stacking (green dashed line), and salt bridges (yellow dashed line) (**a**); Proposed binding mode of atranorin with AChE (PDB id: 4BDT). The key interactions of atranorin with residues in the active sites of AChE: hydrophobic interactions (grey dashed lines), hydrogen bonds (blue solid lines), and π-stacking (green dashed line) (**b**).

As shown in Figure 10a,b, evernic acid and atranorin were able to bind to the active pocket of AChE. Regarding evernic acid, the proposed binding mode displays hydrogen bonds with Thr79, Gly116, and Tyr129; hydrophobic interactions with Trp82; a π-stacking interaction with Trp82 and Tyr333; and a salt bridge interaction with His443 with a binding energy of −11.97 kcal/mol.

The hydrogen bonds between atranorin and AChE were shown to involve Thr79, Trp82, Gly116, Tyr120, Tyr129, Tyr333, Trp435, and Tyr445; hydrophobic interactions with Asp70, Trp82, and Tyr333; and a π-stacking interaction with Trp82, with a binding energy of −13.22 kcal/mol.

In addition, the docking scores for evernic acid and atranorin (binding energies −11.97 kcal/mol and −13.22 kcal/mol, respectively) have statistically significant differences (*p* ≤ 0.05). Obtained results suggest that atranorin is a more active compound against AChE than evernic acid. This result is in accordance with our in vitro study. Thus, atranorin may be an interesting substance for further research aimed at finding new inhibitors of this enzyme.

#### 2.4.3. Butyrylcholinesterase (BChE)

**Figure 11:**
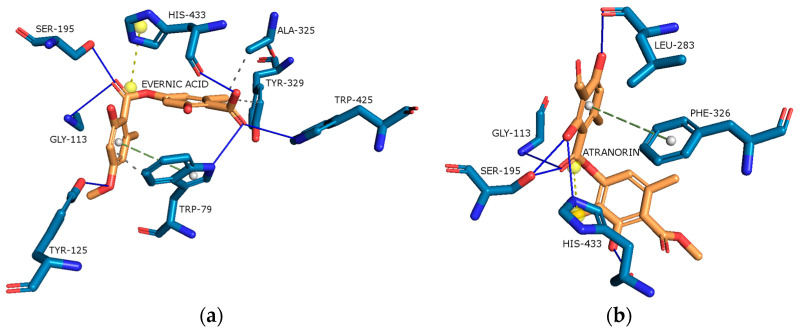
Proposed binding mode of evernic acid with BChE (PDB id: 4BDS). The key interactions of evernic acid with residues in the active sites of BChE: hydrophobic interactions (grey dashed lines), hydrogen bonds (blue solid lines), π-stacking (green dashed line), and salt bridges (yellow dashed line) (**a**). Proposed binding mode of atranorin with BChE (PDB id: 4BDS). The key interactions of atranorin with residues in the active sites of BChE: hydrogen bonds (blue solid lines), π-stacking (green dashed line), and salt bridges (yellow dashed line) (**b**).

As shown in Figure 11a,b evernic acid and atranorin were able to bind to the active pocket of BChE. A high agreement confirmed this was obtained, as the best-docked pose (binding energy of −10.67 kcal/mol and −11.47 kcal/mol, respectively) showed important binding features, among other hydrophobic and hydrogen interactions (see Figure 11a,b). Regarding evernic acid, the proposed binding mode displays hydrogen bonds with Trp79, Gly113, Tyr125, Ser195, Trp425, and His433; hydrophobic interactions with Trp79, Ala325, Tyr329; a π-stacking interaction with Trp79; and a salt bridge interaction with His433.

Hydrogen bonds between atranorin and BChE were shown involving Gly113, Ser195, Leu283, and His433; π-stacking interaction with Phe326; and a salt bridge interaction with His433.

In addition, the docking scores for evernic acid and atranorin (binding energies −10.67 kcal/mol and −11.47 kcal/mol, respectively) do not have statistically significant differences (*p* ≤ 0.05). The obtained results suggest that the inhibition of BChE by evernic acid and atranorin is very close. Both in silico and in vitro studies confirmed the inhibitory effect of both tested depsides on BChE. Therefore, it can be concluded that evernic acid and atranorin could be new interesting anti-BChE compounds.

#### 2.4.4. Tyrosinase (Tyr)

**Figure 12:**
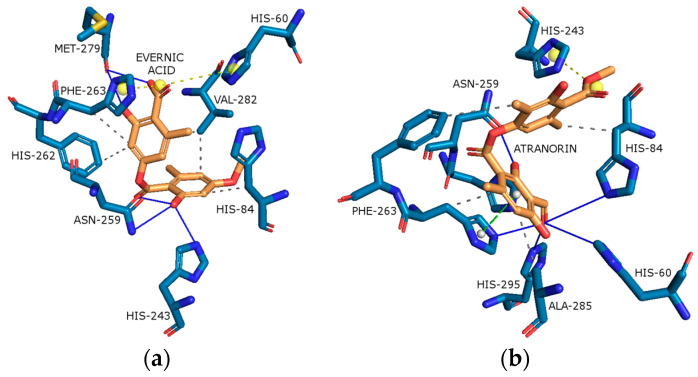
Proposed binding mode of evernic acid with tyrosinase (PDB id: 2Y9X). The key interactions of evernic acid with residues in the active sites of tyrosinase: hydrophobic interactions (grey dashed lines), hydrogen bonds (blue solid lines), and salt bridges (yellow dashed line) (**a**). Proposed binding mode of atranorin with tyrosinase (PDB id: 2Y9X). The key interactions of atranorin with residues in the active sites of tyrosinase: hydrophobic interactions (grey dashed lines), hydrogen bonds (blue solid lines) π-stacking (green dashed line), and salt bridges (yellow dashed line) (**b**).

Evernic acid anchored itself inside the active site gorge of tyrosinase (Figure 12a) by forming a hydrophobic interaction with residues such as His84, His262, Phe263, and Val 282. It also creates hydrogen interactions with residues such as His243, ASN259, and Met279 as well as salt bridge interactions with His60 and His262 with a binding energy of −9.49 kcal/mol.

As shown in Figure 12b, atranorin has several bonding interactions with the tyrosinase active pocket. The best docking score displays hydrogen bonds with His60, His84, Asn259, Phe263, and His295; hydrophobic interactions with His84, His243, Phe263, and Ala285; π-stacking interaction with Phe263; and a salt bridge interaction with His243.

In addition, the docking scores for evernic acid and atranorin (binding energies −9.49 kcal/mol and −10.42 kcal/mol, respectively) do not have statistically significant differences (*p* ≤ 0.05). Obtained results suggest that compounds have the same activity against Tyr. The results of the in silico study are very near to our in vitro measurements, where the inhibition of Tyr by evernic acid was only a bit higher than by atranorin.

#### 2.4.5. α-Glucosidase

**Figure 13:**
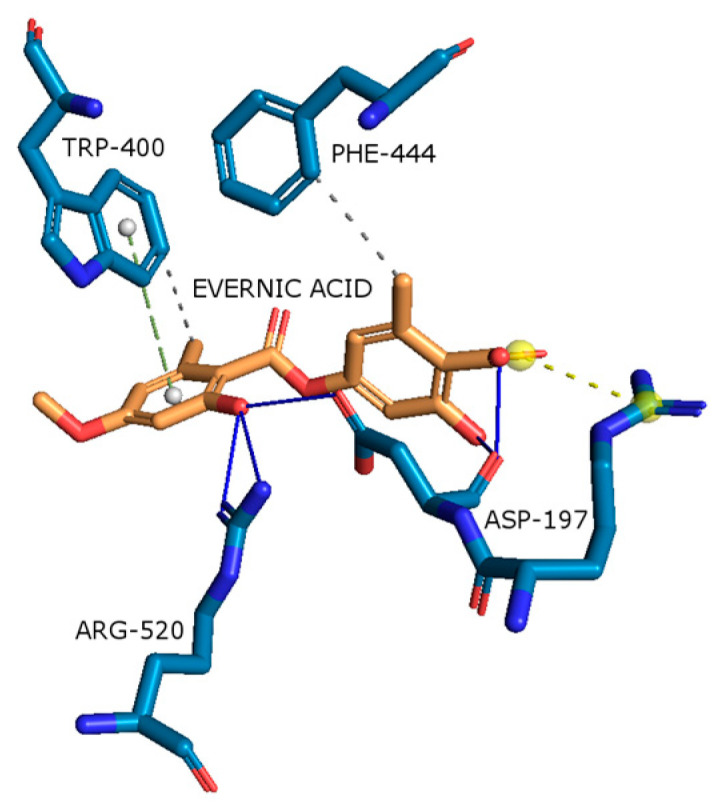
Proposed binding mode of evernic acid with α-glucosidase (PDB id: 2QMJ). The key interactions of evernic acid with residues in the active sites of α-glucosidase: hydrophobic interactions (grey dashed lines), hydrogen bonds (blue solid lines), π-stacking (green dashed line), and salt bridges (yellow dashed line).

Based on the binding energy of the best-docked pose of evernic acid and atranorin in the active site of α-glucosidase, it is indicated that evernic acid is active (negative value) and atranorin is inactive (positive value) against this enzyme (see Table S1). As shown in Figure 13, evernic acid has several bonding interactions with the α-glucosidase active pocket. Evernic acid forms hydrogen bonds with residues such as Asp197 and Arg520, hydrophobic interactions with residues like Phe444 and Trp400, a π-stacking interaction with Trp400, and salt bridge interactions with Asp197, resulting in a binding energy was −8.66 kcal/mol. The in vitro study confirmed the high inhibitory effect of evernic acid on α-glucosidase, in opposition to the very low impact of atranorin.

### 2.5. Permeability through the Blood–Brain Barrier (PAMPA-BBB)

The effective treatment of CNS diseases heavily relies on the active substance’s ability to penetrate the blood–brain barrier. This barrier separates the brain tissues from the rest of the body and protects the brain against harmful substances present in the blood. However, the barrier that safeguards the CNS also impedes access to drugs necessary for dangerous therapies [98].

As part of our research, we evaluated the ability of evernic acid and atranorin in extracts of different polarities to penetrate through a model system of BBB membranes. In vitro tests confirmed our previously published data that lichen-derived depsides can passively penetrate the blood–brain barrier. We have proven that the ability to penetrate is independent of the chemical surroundings, which varies depending on the type of tested extract. The *Pe* × 10^−6^ cm/s parameter, calculated for individual extracts, ranged from 0.74 ± 0.18 to 4.92 ± 0.34 for evernic acid and from 1.95 ± 0.06 to 3.4 ± 0.13 for atranorin (Table 9). It is according to the adopted criteria (the compound was classified as low permeable where *Pe* < 1.5 × 10^−6^ cm/s and high permeable where *Pe* > 1.5 × 10^−6^ cm/s) that the ability of good penetration of the BBB for evernic acid and atranorin can be proved. The lowest result was obtained for the MeOH-H_2_O extract, which was followed by a very low content of evernic acid in this extract.

Limited data are available in the literature on the ability of lichen-derived substances to cross the blood–brain barrier. However, several studies have shown that evernic acid [21], physodic acid [57], usnic acid [21], and caperatic acid [99] were characterized by a high *Pe* coefficient in in vitro tests. This suggests that they have the ability to penetrate the brain tissue area. Moreover, these substances penetrated regardless of the type of tested samples (extracts or pure compounds). Considering the obtained results, the extracts may serve as a natural carrier for active molecules that affect the CNS.

## 3. Materials and Methods

### 3.1. Plant Material and Reagents

The examined lichen (*E. prunastri*) was manually collected from the maple bark, West Pomeranian, XI 2015, and authenticated by Dr Daria Zarabska-Bożejewicz. A voucher specimen (EPES 2015.11) was deposited in the herbarium of the Department of Pharmacognosy and Biomaterials at Poznan University of Medical Sciences.

Sodium carbonate, sodium hydroxide, DMSO, acetone, dichloromethane, methanol, ammonium acetate, and copper (II) chloride were purchased from Avantor Performance Materials Poland S.A. (Gliwice, Poland). The Folin-Ciocalteu phenol reagent was from Merck (Darmstadt, Germany). Evernic acid and (+)-usnic acid were isolated and identified in the Department of Pharmacognosy of Poznan University of Medical Science; atranorin was purchased from ChromaDex (Longmont, CO, USA). All other chemicals were from the Sigma–Aldrich Chemical Co. (Taufkirchen, Germany).

### 3.2. Preparation of Extract

The extracts were prepared from dried crushed thallus of the *E. prunastri*. The precisely weighed amounts of the thallus were extracted four times in an ultrasonic bath, each time for 20 min at a temperature of ~40 °C, using 100 mL of fresh portions of the solvent each time (hexane, dichloromethane (DCM), acetone (Ace), methanol (MeOH), methanol-water 1:1 *v*/*v* (MeOH-H_2_O), and water (H_2_O)). The extracts were filtered and concentrated to dryness or freeze-dried and then used for further studies.

### 3.3. HPLC Analyses of the Extracts

The quantification of *E. prunastri* extracts was effectuated using the HPLC method described by Studzińska-Sroka et al. (2021) with some modifications [57]. The extracts were dissolved in MeOH at a concentration from 0.25 mg/mL to 1.0 mg/mL (a higher concentration was used for the polar extracts). Analysis was performed on the Thermo Scientific UltiMate 3000 UHPLC system. It involved the use of the Kinetex C18 column (100 × 2.1 mm, 5 μm) with a flow rate of 0.5 mL/min and a 10 μL injection volume. The mobile phase consisted of acetonitrile and 0.5% formic acid. The gradient elution increased from 5% of acetonitrile to 100% within 10 min and then the isocratic elution with 100% acetonitrile was proceeded for 2 min. After that, the concentration of acetonitrile was decreasing to initial conditions (5%). The determination was carried out at the wavelength of 254 nm. The results are presented as the average of three independent measurements ± standard deviation (SD). The method was validated for evernic acid, (+)-usnic acid, and atranorin.

### 3.4. Total Phenolic Content (TPC)

The analysis was carried out according to Studzińska-Sroka et al. (2021) [21]. In a 96-well plate, 25.0 µL of the test extract (prepared at concentration 0.2 mg/mL) or 25.0 µL of gallic acid solution (prepared at concentrations 62.5–200 µg/mL) were added, followed by 200 µL of distilled water, 15 µL of Folin-Ciocalteu reagent, and 60 µL of 20% aqueous sodium (IV) carbonate. A blank was created by adding 25.0 µL of DMSO instead of the test extract/gallic acid solution. The plate was covered with aluminum foil to block out light, agitated at 600 RPM for 5 min at room temperature, and then incubated for 25 min without agitation under the same temperature conditions. After incubation, the absorbance was read using a plate reader at 760 nm wavelength (spectrophotometer UV/VIS, Lambda 35, Elmer–Perkin). The experiment was carried out in duplicate and the results are the average of n = 6 measurements. TPC was expressed as mg gallic acid equivalent (GAE) per g of a dry extract ± SD.

### 3.5. Antioxidant Activity

#### 3.5.1. DPPH and CUPRAC Analysis

##### DPPH Analysis

The analysis was carried out according to Studzińska-Sroka et al. (2021) [21]. To the plate, 25 µL of the tested sample (extracts or compounds) and 175 µL of DPPH radical solution were added. The control was created by mixing DPPH radical solution with the solvent used to make dilutions of the tested substance. The blank was determined by measuring the absorbance after mixing 25 µL of the medium in which the tested sample was dissolved. A control test, which was performed simultaneously, contained 25 µL of the extract or anthraquinone solutions to check if the tested substance showed their own absorbance at the tested wavelength. Then, the plate was wrapped in aluminum foil and shaken for 5 min at 25 °C, (RPMI 600). For each concentration of the extract, the number of measurements was n = 6, and for the reference (Vitamin C) n = 3. The incubation time after shaking the plate was 25 min. The absorbance was measured at a 517 nm wavelength. The ability of the standard solutions and extracts from *E. prunastri* to reduce the DPPH radical was calculated using the following formula:

DPPH• scavenging ability (%) = [(A_0_ − A_1_)/A_0_] × 100%

where A_0_ is the absorbance of the control and A_1_ is the absorbance of the tested sample.

The IC_50_ values, or the concentration of antioxidant needed to reduce the initial DPPH• quantity by half, were used to compare the strength of antioxidant properties of the studied extracts. A lower absorbance of the reaction mixture indicated a stronger free radical scavenging activity.

##### CUPRAC Analysis

The analysis was carried out according to Studzińska-Sroka et al. (2021) [21]. To prepare the CUPRAC reagent, equal volumes of neocuproine solution, copper chloride, and ammonium acetate buffer were blended in an aluminum foil-wrapped cone-shaped flask. In total, 50 µL of the extract or standard substance solutions were added to the wells, followed by 150 µL of the test reagent. The control consisted of 50 µL of the solvent for the substance or extract and 150 µL of the test reagent. The plate was covered in aluminum foil and agitated for 5 min and then incubated for an additional 25 min. The experiment was conducted at room temperature. The absorbance was measured at 450 nm wavelength. For each concentration of the extract, the number of measurements was n = 6, and for the reference (Vitamin C) n = 3. Results are presented as IC_0.5_, being the concentration of the tested extract or standard substance at which the absorbance is equal to 0.5.

#### 3.5.2. Chelating Activity of Fe^2+^ and Cu^2+^

##### Fe^2+^ Chelating Activity

The chelating abilities of iron were assessed using a method described in the literature [100]. The experiment was conducted on a 96-well plate, where 200 μL of the extract being tested or 200 μL of a standard substance solution (quercetin) at a chosen concentration and 10 μL of an iron chloride (II) aqueous solution were added to the test wells. The control mixture consisted of 200 μL DMSO and 10 μL of an iron chloride (II) aqueous solution. The plate was shaken for 10 min at room temperature using a shaker set at 500 RPM. After the first incubation stage, 40 μL of a ferrozine aqueous solution was added to the test samples and the control sample. Distilled water was added instead of ferrozine for both test and control blanks. The samples were then incubated for an additional 10 min using the shaker at 500 RPM and at room temperature. The plate was covered with aluminum foil to protect it from light at all times. Finally, the absorbance at a wavelength of 562 nm was measured using a plate reader (Multiskan GOx1510 from Thermo-Scientific, Waltham, MA, USA) after the incubation period. For the investigated substances, two independent experiments were carried out and the average from n = 4 measurements was calculated. The percentage of inhibition was determined using the following equation:

$${Fe}^{2+}chelating\;activity\left[\%\right]=[1-\frac{\left(As-Asb\right)}{\left(Ac-Acb\right)}]\times 100$$

where As is the absorbance of the sample, Asb is the absorbance of the blank of the sample, Ac is the absorbance of the control, and Acb is the blank of the control.

##### Cu^2+^ Chelating Activity

The chelating abilities of iron were assessed using a method described in the literature [101]. In the test tubes, 30 μL of the extract being tested or a standard substance solution at a chosen concentration, 175 μL of a buffer solution made of sodium acetate, and 30 μL of CuSO_4_•5H_2_O solution were added to the test wells. The control mixture was prepared in the wells by adding 30 μL of DMSO (the medium used to dissolve the substance being tested) and the remaining reagents were the same as the test samples. The plate was then shaken for 10 min using a shaker (500 RPM) at room temperature. After the first incubation stage, 15 μL of a pyrocatechol violet (PV) solution was added to both the test samples and the control sample. For the test and control blanks, the PV solution was substituted with 15 μL of a sodium acetate buffer solution. The plate was incubated again for 20 min using a shaker (500 RPM) at room temperature. The plate was covered with aluminum foil to block light. The absorbance was measured at a wavelength of 632 nm after the incubation period. For the investigated substances, two independent experiments were carried out and the average from n = 6 for extracts and n = 4 for quercetin measurements was calculated. The percentage of inhibition was determined using the following equation:

$${Cu}^{2+}chelating\;activity\left[\%\right]=[1-\frac{\left(As-Asb\right)}{\left(Ac-Acb\right)}]\times 100$$

where As is the absorbance of the sample, Asb is the absorbance of the blank of the sample, Ac is the absorbance of the control, and Acb is the blank of the control.

#### 3.5.3. Enzymatic Activity

##### Effect on Superoxide Dismutase Activity (SOD)

Inhibitory activity on superoxide dismutase activity (SOD) was evaluated as described previously [21], except that the samples were dissolved in DMSO at a concentration of 1 mg/mL.

##### Effect on Superoxide Dismutase Activity (GR)

Inhibitory activity on glutathione reductase (GR) was evaluated as described previously [21], except that the samples were dissolved in DMSO at a concentration of 1 mg/mL.

##### Effect on Glutathione Peroxidase (GPx)

Inhibitory activity on glutathione peroxidase (GPx) was evaluated as described previously [21], except that the samples were dissolved in DMSO at a concentration of 1 mg/mL.

##### Effect on Catalase Activity (CAT)

The method of Watanabe et al. (2007) was used with the following modifications [102]. The reaction mixture was composed of 0.02 mL EDTA solution (56.5 mmol/dm^3^), 0.01 mL sample, 0.02 mL 3% H_2_O_2_ solution (Sigma H1009), and 0.02 mL catalase solution (4000-fold diluted, Sigma C3515) (all reagents except tested sample were diluted in TRIS buffer, pH 7.0, 1 mol/dm^3^). The volume was completed to 0.31 mL by the same buffer solution. In a blank sample, DMSO (Sigma D4540) replaced the tested sample. The background of the sample was measured in a mixture composed of 0.01 mL sample completed to 0.31 mL by buffer. The absorbance was read at 240 nm (Varioskan Lux, Thermo Scientific) directly after the mixing and after 5 min of incubation (room temperature). The decrease in absorbance (depletion of H_2_O_2_) in the tested and blank sample was compared. The calibration curve was produced using eleven H_2_O_2_ solutions (0.5693–5.693 mmol/dm^3^). The results are expressed in terms of % inhibition as well as in the decrease in H_2_O_2_ depletion (mmol/dm^3^ min reaction).

### 3.6. Anti-Inflammatory Activity

#### 3.6.1. Effect on Cyclooxygenase-2 Activity (COX-2)

The inhibitory effect on cyclooxygenase-2 activity (COX-2) was evaluated using a Cayman COX Activity Assay Kit (No. 760151) as described previously [21], except that the samples were dissolved in DMSO at a concentration of 1 mg/mL.

#### 3.6.2. Effect on Hyaluronidase Activity

Hyaluronidase inhibition was assessed using a method described in the literature [70]. In summary, the following was performed: 25 µL of incubation buffer (50 mM, pH 7.0, with 77 mM NaCl and 1 mg/mL albumin), 25 µL enzyme (30 U/mL in incubation buffer), 10 µL of tested extracts (of 3.125, and 6.25 mg/mL), and 15 µL acetate buffer (pH 4.5) were mixed. The mixture was incubated at 37 °C for 15 min, after which 25 µL of hyaluronic acid (HA) (0.3 mg/mL in acetate buffer) was added and incubated for an additional 45 min at 37 °C. Then, 200 µL of 2.5% CTAB in 2% NaOH was added and the reaction mixture’s turbidity was measured as the absorbance at 600 nm (using a Multiskan GOx1510 from Thermo-Scientific) after 10 min of incubation at room temperature. β-escin was used as a positive control at concentrations of 6.25 mg/mL. This procedure was repeated 2 times and the average of n = 3 measurements was taken. The percentage of inhibition was determined using the following equation:

$$Hyaluronidase\;inhibition\left[\%\right]=\frac{\left(T_{s}-{TE}_{blank}\right)}{{(TH}_{blank}-{TE}_{blank})}\times 100\%$$

where T_S_—absorbance of sample; TE_blank_—absorbance of the enzyme + examined substance; and TH_blank_—absorbance of the HA + examined substance.

### 3.7. Neuroprotective Activity

#### 3.7.1. Effect on Acetylcholinesterase (AChE) and Butyrylcholinesterase (BChE) Activity

Cholinesterase activities were assessed using a method described in the literature with modification [103]. In the test tubes, 5 μL of either the tested at 40 mg/mL (DCM, Ace, MeOH, MeOH-H_2_O, H_2_O extracts, and EA) or 10 mg/mL (Hex extract and ATR—very low solubility at 40 mg/mL, EA) was added, along with 60 μL of Tris-HCl buffer solution and 30 μL of AChE or BChE enzyme solution. The control test tubes were given 5 μL of DMSO and all other reagents were the same as in the tested samples. The blank samples for the tested and control samples received 30 μL of Tris-HCl buffer solution instead of the enzyme solution. The plate was agitated for 5 min at room temperature with a shaker at 500 RPM. After the initial incubation, all tubes received 30 μL of ATCh or BTCh solution and 125 μL of DTNB solution. The plate was then incubated for an additional 20 min at room temperature with the shaker at 500 RPM, covered in aluminum foil to block out light. At the end of the incubation period, the absorbance at a wavelength of 405 nm was measured using a 96-well microplate reader (Multiskan GOx1510 from Thermo-Scientific). For the investigated substances, two independent experiments were carried out and the average from n = 4 for extracts and n = 3 for lichen metabolites measurements were calculated. The percentage of inhibition was determined using the following equation:

$$AChE\;or\;BChE\;inhibition\left[\%\right]=[1-\frac{\left(As-Asb\right)}{\left(Ac-Acb\right)}]\times 100$$

where As is the absorbance of the sample, Asb is the absorbance of the blank of the sample, Ac is the absorbance of the control, and Acb is the blank of the control.

#### 3.7.2. Effect on Tyrosinase Activity

The extracts from *E. prunastri* and evernic acid were dissolved in DMSO to obtain a concentration of 16 mg/mL and 8 mg/mL. The spectrophotometric method was used [57], with some modifications. Briefly, 25 µL of the sample, 75 µL of 0.02 M phosphate buffer (pH 6.8), and 50 µL of tyrosinase solution (192 U/mL in phosphate buffer) were mixed. Next, the samples were incubated at room temperature (25 °C) for 10 min with shaking (500 rpm). Subsequently, 50 µL of L-DOPA (2 mM in phosphate buffer) was added and incubated for 20 min with shaking (500 rpm) at the same temperature condition (25 °C). The blanks of samples were prepared using 50 µL of the buffer instead of L-DOPA solutions. The control sample contained DMSO instead of the tested substances. The control blank contained 25 µL of DMSO instead of samples and 50 µL of the buffer instead of L-DOPA solution. The azelaic acid (at concentrations 1.6 mg/mL and 0.8 mg/mL, dissolved in DMSO) was used as the reference. Absorbance was measured at 475 nm (Multiskan GO 1510, Thermo Fisher Scientific, Vantaa, Finland). Two independent experiments were carried out for the investigated substances and the average from n = 6 for extracts and azelaic acid and n = 3 for lichen metabolites measurements was calculated. The percentage of tyrosinase inhibition was calculated as follows:

$$Tyrosinase\;inhibition\left[\%\right]=[1-\frac{\left(As-Asb\right)}{\left(Ac-Acb\right)}]\times 100$$

where As is the absorbance of the sample, Asb is the absorbance of the blank of the sample, Ac is the absorbance of the control, and Acb is the blank of the control.

#### 3.7.3. α-Glucosidase Inhibitory Assay

Inhibition of α-glucosidase by the extracts was performed according to Studzińska-Sroka et al. (2021) [100] with some modifications. Briefly, 25.0 μL of extracts solution (1, 3 and 5 mg/mL), 50.0 μL of 0.02 M phosphate buffer (pH 6.8), and 75.0 µL of α-glucosidase solution (0.5 U/mL) were pre-incubated in 96 well plates at 37 °C for 15 min. Next, 50.0 μL of 5 mM p-nitrophenyl-α-D-glucopyranoside (pNPG) solution in 0.02 M phosphate buffer (pH 6.8) was added and incubated at 37 °C for 20 min. The reaction was terminated by adding 100.0 µL of sodium carbonate (0.2 M) into the mixture. The absorbance was measured after 2 min at 405 nm. The acarbose was used as a reference. The control samples showed the total enzyme activity and were prepared with DMSO instead of the examined substance. The blank for the control was prepared without enzyme replacing it with buffer and with DMSO instead of examined substance. The blanks for samples were prepared without enzyme replacing it with buffer. All experiments were carried out two times and the average from n = 3 measurements was calculated. The enzyme inhibition rate (presented for the final concentration of substance in enzymatic reaction) was expressed as a percentage of inhibition and calculated using the following formula:

$$\alpha -Glucosidase\;inhibition\left[\%\right]=[1-\frac{\left(As-Asb\right)}{\left(Ac-Acb\right)}]\times 100$$

where As is the absorbance of the sample, Asb is the absorbance of the blank of the sample, Ac is the absorbance of the control, and Acb is the blank of the control.

### 3.8. Principal Component Analysis (PCA)

Correlations were examined using principal component analysis (PCA) with PQStat Software version 1.8.4.142 and Statistica 13.3.

### 3.9. Molecular Docking on Cholinesterse and Tyrosinase Activity

X-ray crystal structures of COX-2 (5F1A), human AChE (4BDT), human BChE (4BDS), tyrosinase (2Y9X), and α-glucosidase (2QMJ) were retrieved from the Protein Data Bank (PDB) [104] (https://www.rcsb.org/, accessed on 12 November 2022 and 16 November 2023). Molecular structures of the evernic acid (PubChem CID: 10829) and atranorin (PubChem CID: 68066) were downloaded from PubChem [105] (https://pubchem.ncbi.nlm.nih.gov, accessed on 12 November 2022 and 16 November 2023) in sdf format. Prior to molecular docking, the geometries of the evernic acid and atranorin were optimized using Gaussview (Wallingford, CT, USA) software (B3LYP/6-31 (d,p)). Next, the ligands and proteins were prepared using AutoDock Tools (ADT; Scripps Research Institute, La Jolla, San Diego, CA, USA) [106], where water and other ligands are removed and polar hydrogen atoms and Kollman charges are added and saved in pdbqt format. The AutoDock Tools were used to create a grid box to incorporate the entire active site for each protein structure of COX-2, AChE, BChE, tyrosinase, and α-glucosidase (Table 10) and the Lamarckian Genetic Algorithm method with 100 conformations.

The molecular docking was performed by Autodock Vina version 1.2.0 (The Scripps Research Institute, La Jolla, San Diego, CA, USA), an open-source docking program [107,108]. After docking simulations, the best scoring pose was selected and exported to the PDBQT format. The file was converted to the PDB format with the Open Babel program and opened in the protein–ligand interaction profiler (PLIP server, https://plip-tool.biotec.tu-dresden.de/, accessed on 14 November 2022) [109]. Interactions between evernic acid and the active site of the enzymes were determined using the PLIP server. Next, a file in PSE format was downloaded with the PLIP server. The evernic acid–enzyme and atranorin–enzyme docked complexes (file in PSE format) were visualized using the PyMOL tool (DeLano Scientific LLC, Palo Alto, CA, USA) [110]. The docked complexes were later uploaded to Prank-Web [111,112,113] (https://prankweb.cz/, accessed on 24 November 2023), where 3D models were generated and evaluated the active site.

### 3.10. Permeability through the Blood–Brain Barrier (PAMPA-BBB)

To assess the effective permeability (*Pe*) of evernic acid and atranorin contained in *E. prunastri* extracts through the blood–brain barrier (BBB), the parallel artificial membrane permeability assay (PAMPA) for the blood–brain barrier (BBB) was used (Pion Inc., Billerica, MA, USA). Briefly, the donor solution (180 µL) containing the extracts (10 µL of extracts stock solution at concentration 5 mg/mL was diluted with 990 µL of Prisma buffer, pH = 7.4 (Prisma HT, Pion Inc.) was added to the donor wells. Next, each filter membrane of acceptor plate wells was coated with 5 μL BBB-1 lipid solution (Pion Inc.). After a few minutes, 200 µL BSB (Brain Sink Buffer, Pion Inc.) was added to the acceptor plate. Incubation lasted for 4 h at 37 °C. Finally, the evernic acid concentration was verified using the HPLC analysis. The equation below was used to calculate the effective permeability (*Pe*) of lichen-derived compound:

$$P_{e}=-\frac{\ln\left(1-\frac{C_{A}}{C_{eq}}\right)}{S\times \left(\frac{1}{V_{D}}+\frac{1}{V_{A}}\right)\times t}$$

where *Pe* is the effective permeability coefficient (cm/s), V_D_—donor volume, V_A_—acceptor volume, S—membrane area, and t—incubation time (in seconds), and C_eq_—equilibrium concentration $C_{eq}=\frac{C_{D}\times V_{D}+C_{A}\times V_{A}}{V_{D}+V_{A}}$ [114,115].

Two independent experiments were conducted and the average from n = 4 results ± SEM for evernic acid and from n = 2 results ± SEM for atranorin was reported. Compounds with *Pe* (×10^−6^ cm/s) > 1.5 are classified as high permeable the *Pe* (×10^−6^ cm/s) < 1.5 values are classified as low permeable [114,115].

### 3.11. Statistical Analysis

Statistical analysis was performed using GraphPad Prism™ 6.00 software (Graph Pad Software Inc., San Diego, CA, USA). Results were expressed as means ± SD (standard deviation) or SEM (standard error of the mean). The median effect concentrations (IC_50_ or IC_0.5_ values) were determined using a concentration–response curve.

Correlations were examined using principal component analysis (PCA) with PQStat Software version 1.8.4.142 and Statistica 13.3.

## 4. Conclusions

In conclusion, *E. prunastri* is a lichen characterized by multi-directional biological activity. Results presented in the manuscript indicate that the lowest polarity *E. prunastri* extracts contain polyphenols, especially a high level of evernic acid and atranorin. Despite this, they possess moderate antioxidant activity. The high content of atranorin in Hex extract and evernic acid and atranorin in DCM extract, Ace extract, and MeOH extract may influence their properties to inhibit enzymes, associated with AD and PD development (AChE, BChE, and tyrosinase). It is especially noticeable that atranorin has a high ability to inhibit AChE, which was confirmed in in vitro and in silico tests. In contrast, evernic acid and extracts with high evernic acid content (mainly DCM extract, Ace extract, and MeOH extract) show interesting anti-inflammatory (COX-2) and hypoglycemic (α-glucosidase) potential, which was proved using all our analysis. It is important to note that in addition to evernic acid or atranorin, which are important for the biological activity of *E. prunastri* extracts, their biological potential may also depend on the synergistic effect of other compounds, as well as those present in the extracts in low concentrations. In our study, we confirmed that the dominant substance in *E. prunastri* evernic acid and atranorin could penetrate the blood–brain barrier from the extract’s matrix. Although our experiments is preliminary, we suggest that *E. prunastri* and its secondary metabolites, evernic acid and atranorin, are characterized by pleiotropic biological potential, which prompts further investigation in the search for substances to use in treating neurodegenerative diseases.

## Figures and Tables

**Table 1:** Content of evernic acid, usnic acid, and atranorin in *Evernia prunastri* extracts as the results of HPLC analysis.

Content [%]	Hex	DCM	Ace	MeOH	MeOH-H_2_O	H_2_O
Evernic acid	1.81 ± 0.01	63.13 ± 0.31	55.22 ± 1.24	58.32 ± 0.20	23.95 ±0.06	0.11 ± 0.01
Atranorin	61.04 ± 3.45	44.46 ± 0.63	37.70 ± 1.14	19.72 ± 0.16	0.65 ± 0.05	0.53 ± 0.03
Usnic acid	5.25 ± 0.11	1.66 ± 0.03	1.52 ± 0.05	1.02 ± 0.02	1.07 ± 0,08	0.06 ± 0.01

The mean values ± SD from *n* = 3 measurements are presented. Hex—hexane extract, DCM—dichloromethane extract, Ace—acetone extract, MeOH—methanol extract, MeOH-H_2_O—methanol-water extract, H_2_O—water extract.

**Table 2:** The antioxidant activity of *Evernia prunastri* extracts.

Extracts/ Substance	DPPH IC_50_ (mg/mL)	CUPRAC IC_0.5_ (mg/mL)
Hex	>28.00	0.16 ± 0.00
DCM	>28.00	0.47 ± 0.02
Ace	24.31 ± 0.62	0.47 ± 0.03
MeOH	9.37 ± 0.61	0.69 ± 0.02
MeOH-H_2_O	6.83 ± 0.55	1.03 ± 0.03
H_2_O	4.83 ± 1.97	0.61 ± 0.05
Vitamin C	0.074 ± 0.001	0.058 ± 0.001

The mean values ± SD from n = 6 measurements for extracts and n = 3 for reference are presented. DPPH—2,2-Diphenyl-1-picrylhydrazyl assay, CUPRAC—cupric ion reducing antioxidant capacity assay, Hex—hexane extract, DCM—dichloromethane extract, Ace—acetone extract, MeOH—methanol extract, MeOH-H_2_O—methanol-water extract 1:1 *v*/*v*, H_2_O—water extract.

**Table 3:** Fe^2+^ chelating activity of *Evernia prunastri* extracts.

Tested Sample	Chelating Fe^2+^ [%]		
	Concentration [mg/mL]		
	0.5	1.5	2.5
Hex	8.15 ± 2.36	36.79 ± 1.19	75.82 ± 2.45
DCM	na	13.31 ± 3.36	19.91 ± 4.95
Ace	10.94 ± 1.33	14.93 ± 3.65	24.20 ± 2.06
MeOH	12.03 ± 3.15	44.77 ± 3.12	57.30 ± 6.05
MeOH-H_2_O	85.12 ± 5.14	95.57 ± 1.30	97.21 ± 0.24
H_2_O	67.02 ± 0.97	92.13 ± 2.17	98.45 ± 0.49
Quercetin	44.05 ± 2.66	58.95 ± 0.85	79.47 ± 3.14

The mean values ± SD from n = 4 measurements are presented; “na”—not active at this concentration; Hex—hexane extract, DCM—dichloromethane extract, Ace—acetone extract, MeOH—methanol extract, MeOH-H_2_O—methanol-water extract 1:1 *v*/*v*, H_2_O—water extract.

**Table 4:** Cu^2+^ chelating activity of *Evernia prunastri* extracts.

Tested Sample	Chelating Cu^2+^ [%]				
	Concentration [mg/mL]				
	0.1	0.5	1.5	2.5	5
Hex	10.96 ± 2.35	59.16 ± 1.17	90.06 ± 1.91	95.17± 4.16	87.70± 6.22
DCM	5.69 ± 0.92	35.02 ± 0.60	59.44 ± 3.09	74.89 ± 1.91	89.56 ± 2.20
Ace	10.12 ± 3.74	41.73 ± 6.09	61.70 ± 9.56	77.22 ± 7.33	88.78 ± 3.67
MeOH	6.95 ± 2.68	31.99 ± 3.50	66.46 ± 7.33	84.15 ± 4.75	94.48 ± 1.24
MeOH-H_2_O	1.86 ± 1.53	18.39 ± 1.90	43.84 ± 1.08	62.24 ± 1.81	82.81 ± 1.32
H_2_O	3.09 ± 2.69	32.38 ± 3.60	68.43 ± 3.80	82.44 ± 1.92	91.63 ± 1.48
Quercetin	53.16 ± 2.24	97.57 ± 0.98	nt	nt	nt

The mean values ± SD from n = 6 measurements for extracts and n = 4 for quercetin are presented; “nt”—not tested; Hex—hexane extract, DCM—dichloromethane extract, Ace—acetone extract, MeOH—methanol extract, MeOH-H_2_O—methanol-water extract 1:1 *v*/*v*, H_2_O—water extract.

**Table 5:** Effect of *Evernia prunastri* extracts and evernic acid on SOD, GR, GPx, and CAT activity.

Tested Sample	SOD Inhibition (%)	GR Inhibition (%)	GR Inhibition (nmol NADPH/ min Incubation)	GPx Inhibition (%)	GPx Inhibition (nmol NADPH/ min Incubation)	CAT Inhibition (%)	CAT Inhibition of the H_2_O_2_ Depletion (mmol/mL min Reaction)
Hex	37.4 ± 2.6	14.9 ± 5.1	559 ± 191	40.0 ± 5.6	79.6 ± 11.1	13.5 ± 3.9	0.03 ± 0.01
DCM	35.6 ± 1.6	22.4 ± 4.2	840 ± 158	29.7 ± 4.3	59.1 ± 8.6	74.0 ± 3.5	0.36 ± 0.02
Ace	35.0 ± 1.0	24.2 ± 3.2	907 ± 120	16.4 ± 3.2	32.6 ± 6.4	na	na
MeOH	50.1 ± 2.3	36.3 ± 2.9	1374 ± 110	54.0 ± 2.6	107.5 ± 5.2	na	na
MeOH -H_2_O	48.0 ± 2.7	37.2 ± 3.0	1396 ± 113	26.7 ± 3.4	53.1 ± 6.8	18.6 ± 4.0	0.07 ± 0.02
H_2_O	38.9 ± 3.6	26.9 ± 4.6	1011 ± 173	39.2 ± 2.4	78.0 ± 4.8	18.3 ± 4.5	0.07 ± 0.02
EA	34.1 ± 1.7	10.3 ± 3.7	388 ± 139	10.0 ± 2.6	19.9 ± 5.2	na	na

The mean values ± SD are presented; “na” –not active; Hex—hexane extract, DCM—dichloromethane extract, Ace—acetone extract, MeOH—methanol extract, MeOH-H_2_O—methanol-water extract, H_2_O—water extract.

**Table 7:** Effect of *Evernia prunastri* extracts and evernic acid on α-glucosidase activity.

Tested Sample	α-Glucosidase Inhibition [%]			
	1 mg/mL	3 mg/mL		5 mg/mL
Hex	14.23 ± 2.13	80.69 ± 5.94		94.18 ± 3.75
DCM	5.74 ± 2.50	17.55 ± 0.83		98.33 ± 2.00
Ace	3.64 ± 0.56	15.64 ± 2.07		97.13 ± 2.51
MeOH	0.13 ± 0.11	11.35 ± 3.29		84.03 ± 5.78
MeOH-H_2_O	na	2.63 ± 1.25		4.66 ± 0.43
H_2_O	na	na		3.19 ± 1.86
EA	nt	nt		95.00 ± 6.06
ATR	nt	nt		19.14 ± 8.50
Acarbose	10 mg/mL		50 mg/mL	
	1.59 ± 8.12		85.23 ± 8.12	

The mean values ± SD from n = 3 measurements are presented “na”—not active; “nt”—not tested; Hex—hexane extract, DCM—dichloromethane extract, Ace—acetone extract, MeOH—methanol extract, MeOH-H_2_O—methanol-water extract, H_2_O—water extract, EA—evernic acid, ATR—atranorin.

**Table 8:** The molecular interaction between the enzyme* (grey) and compound (orange). The active site of the enzyme predicted by PrankWeb is colored blue or red. *—due to software limitations, simulations for tyrosinase were not presented in this table.

	Evernic Acid	Atranorin
COX-2	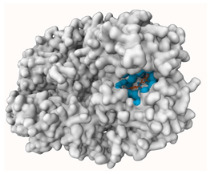	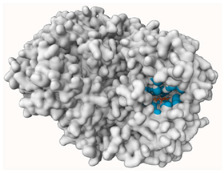
AChE	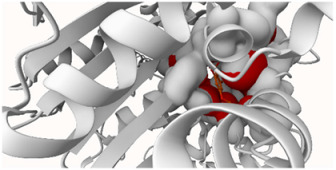	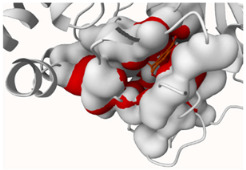
BChE	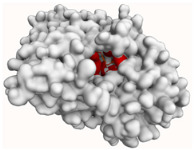	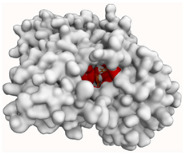
α-Glucosidase	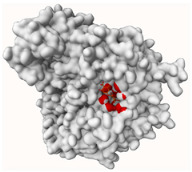	na

“na”—not active.

**Table 9:** The effective permeability (*Pe*) of evernic acid as pure compounds and from extracts using the parallel artificial membrane permeability assay for the blood–brain barrier (PAMPA-BBB).

Tested Sample	*Pe* × 10^−6^ [cm/s] (t = 4 h)	Tested Sample	*Pe* × 10^−6^ [cm/s] (t = 4 h)
Evernic acid (from Hex)	2.63 ± 1.50	Atranorin (from Hex)	3.07 ± 0.13
Evernic acid (from DCM)	3.16 ± 0.94	Atranorin (from DCM)	1.95 ± 0.06
Evernic acid (from Ace)	2.20 ± 0.73	Atranorin (from Ace)	2.85 ± 0.14
Evernic acid (from MeOH)	4.92 ± 0.34	Atranorin (from MeOH)	3.40 ± 0.13
Evernic acid (from MeOH-H_2_O)	0.74 ± 0.18	Atranorin (from MeOH-H_2_O)	nd
Evernic acid (from H_2_O)	nd	Atranorin (from H_2_O)	nd
Evernic acid	8.6 ± 0.4 *	Atranorin	2.2 ± 0.1 **

“nd”—not determined; “*”—the value taken from the literature [21]; “**”—the value taken from the literature [99]. Results are presented as *Pe* × 10^−6^ cm/s. The mean values of four measurements (n = 4) ± SEM (evernic acid) or of two measurements (n = 2) ± SEM are presented. Hex—hexane extract, DCM—dichloromethane extract, Ace—acetone extract, MeOH—methanol extract, MeOH-H_2_O—methanol-water extract, H_2_O—water extract.

**Table 10:** Parameters used to create a grid box in the case of COX-2, AChE, BChE, tyrosinase, and α-glucosidase.

	PDB Code	Coordinates of Grid Box	Size of Grid Box (Å)	Maximum Radius Limit (Å)
COX-2	5F1A	x = 30.725 y = 35.102 z = 242.610	x = 52 y = 62 z = 60	0.375
Human AChE	4BDT	x = −1.18 y = −36.63 z = −51.58	x = 40 y = 40 z = 40	0.375
Human BChE	4BDS	x = 136.26 y = 115.98 z = 42.30	x = 40 y = 40 z = 40	0.375
Tyrosinase	2Y9X	x = −8.064 y = −25.776 z = −39.384	x = 60 y = 60 z = 60	0.375
α-glucosidase	2QMJ	x = −20.83 y = −6.56 z = −5.04	x = 40 y = 40 z = 40	0.375

## Data Availability

The data supporting reported results can be found in the Department of Pharmacognosy and Biomaterials, Poznan University of Medical Sciences and in the Department of Biotechnology, Microbiology and Human Nutrition, University of Life Sciences in Lublin.

## Supplementary Materials

The following supporting information can be downloaded at: https://www.mdpi.com/article/10.3390/molecules29010233/s1, Figure S1: The chromatogram of hexane extract from *E. prunastri*, showing the identified compounds, i.e., evernic acid, atranorin and (+)-usnic acid, respectively; Figure S2: The chromatogram of methanol-water extract from *E. prunastri*, showing the identified compounds, i.e., evernic acid (6.997 min), atranorin (8.430 min) and (+)-usnic acid (8.280), respectively; Figure S3: The chromatogram of water extract from *E. prunastri*, showing the identified compounds, i.e., evernic acid (7.010 min), atranorin (8.430 min) and (+)-usnic acid (8.280 min), respectively; Table S1: Molecular docking of evernic acid and atranorin with various drug target enzymes.
