# Supplementary material for: Pleiotropic Potential of Evernia prunastri Extracts and Their Main Compounds Evernic Acid and Atranorin: In Vitro and In Silico Studies
Source: Molecules. 2023 Dec 31;29(1):233. doi: 10.3390/molecules29010233 (PMC10780513; doi:10.3390/molecules29010233)
Supplement: Supplementary file 1 [file molecules-29-00233-s001.zip › molecules-2776689-supplementary.pdf]

molecules

MDPI

Supplementary Materials

Pleiotropic Potential of *Evernia prunastri* Extracts and Their Main Compounds Evernic Acid and Atranorin: In Vitro and In Silico Studies

Elżbieta Studzińska-Sroka [1,*], Magdalena Bulicz [1], Marika Henkel [1], Natalia Rosiak [1], Magdalena Paczkowska-Walendowska [1], Dominik Szwajgier [2], Ewa Baranowska-Wójcik [2], Katarzyna Korybalska [3] and Judyta Cielecka-Piontek [1]

[1] Department of Pharmacognosy and Biomaterials, Poznan University of Medical Sciences, Rokietnicka 3 Str., 60-806 Poznań, Poland
[2] Department of Biotechnology, Microbiology and Human Nutrition, University of Life Sciences in Lublin, Skromna 8 Str., 20-704 Lublin, Poland
[3] Department of Patophysiology, Poznan University of Medical Science, Rokietnicka 8 Str., 60-806 Poznań, Poland

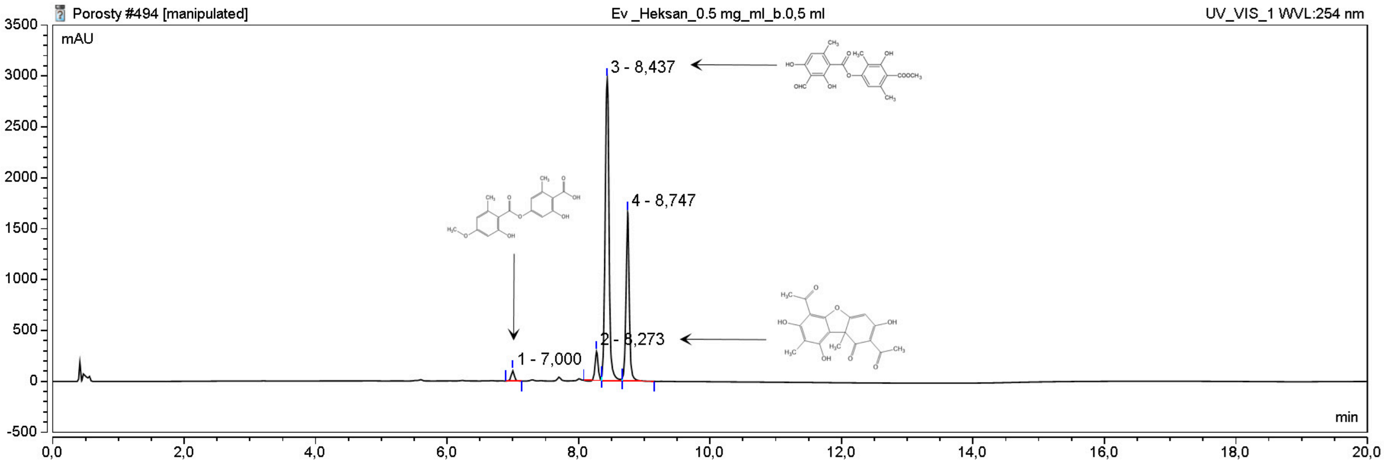

Figure S1. The chromatogram of hexane extract from *E. prunastri*, showing the identified compounds, i.e., evernic acid, atranorin and (+)-usnic acid, respectively.

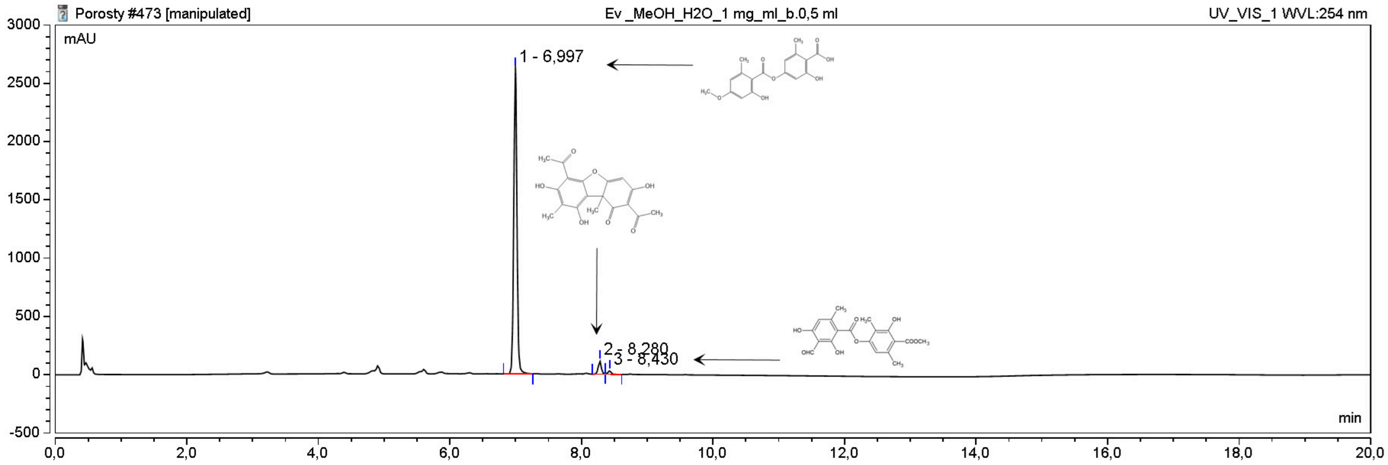

Figure S2. The chromatogram of methanol-water extract from *E. prunastri*, showing the identified compounds, i.e., evernic acid (6.997 min), atranorin (8.430 min) and (+)-usnic acid (8.280), respectively.

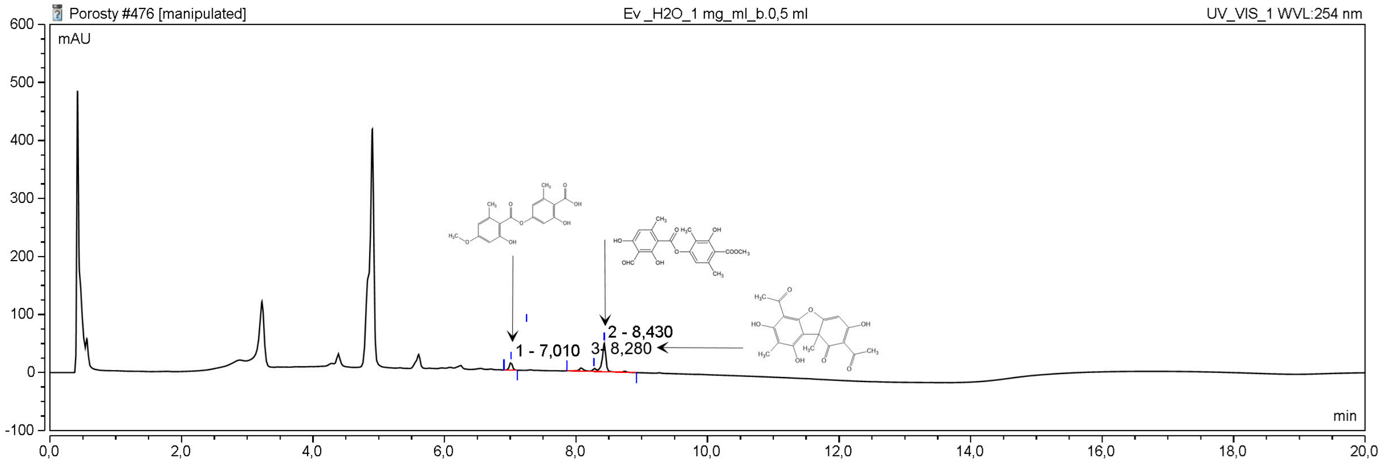

Figure S3. The chromatogram of water extract from *E. prunastri*, showing the identified compounds, i.e., evernic acid (7.010 min), atranorin (8.430 min) and (+)-usnic acid (8.280 min), respectively.

Table S1. Molecular docking of evernic acid and atranorin with various drug target enzymes

**Target molecule**	PDB code	Binding Energy [kcal/mol]		K_i [nM]		Receptor contact	
		evernic acid	atranorin	evernic acid	atranorin	evernic acid	atranorin
**COX-2***	5F1A	-11.85	- 12.47	2.08	0.72	ALA168<br>GLN172<br>PHE179<br>THR181<br>ASN351<br>HIS355<br>TRP356<br>HIS357<br>LEU360	ALA168<br>HIS176<br>PHE179<br>THR181<br>ASN351<br>TYR354<br>HIS355<br>TRP356<br>HIS357<br>LEU359
AChE*	4BDT	−11.97	−13.22	1.69	0.21	THR79<br>TRP82<br>GLY116<br>TYR129<br>TYR333<br>HIS443	ASP70<br>THR79<br>TRP82<br>GLY116<br>TYR120<br>TYR129<br>TYR333<br>TRP435<br>TYR445
BChE	4BDS	−10.67	−11.47	15.07	3.92	TRP79<br>GLY113<br>TYR125<br>SER195<br>ALA325<br>TYR329<br>TRP425<br>HIS433	GLY113<br>SER195<br>LEU283<br>PHE326<br>HIS433
Tyrosinase	2Y9X	−9.49	−10.42	110.47	23.01	HIS60<br>HIS84<br>HIS243	HIS60<br>HIS84<br>HIS243

						ASN259<br>HIS262<br>PHE263<br>MET279<br>VAL282	ASN259<br>PHE263<br>ALA285<br>HIS295
α-glucosidase*	2QMJ	−8.66	+51.81	448.87	na	ASP197<br>TRP400<br>PHE444<br>ARG520	na

K_i – estimated inhibition constant, "na" – not active *– indicates statistically significant differences *(*p*≤0.05)* in enzyme activity (the significance determined based on the comparison of binding energies).
